# C-reactive protein and cardiovascular risk in the general population

**DOI:** 10.1093/eurheartj/ehaf937

**Published:** 2025-12-11

**Authors:** Berkan Kurt, Martin Reugels, Kai M Schneider, Jens Spiesshoefer, Andrea Milzi, Alexander Gombert, Christopher B Fordyce, Florian A Wenzl, Neha J Pagidipati, Viviane Rocha, Marat Fudim, Abhinav Sharma, Michael Lehrke, Hiroaki Shimokawa, Giovanna Liuzzo, Lale Tokgozoglu, Filippo Crea, Thomas F Lüscher, Peter Libby, Paul M Ridker, Nikolaus Marx, Carolin V Schneider, Florian Kahles

**Affiliations:** Department of Internal Medicine I—Cardiology, University Hospital Aachen, RWTH Aachen University, Pauwelsstraße 30, Aachen D-52074, Germany; Department of Internal Medicine I—Cardiology, University Hospital Aachen, RWTH Aachen University, Pauwelsstraße 30, Aachen D-52074, Germany; Department of Internal Medicine III—Gastroenterology, University Hospital Aachen, RWTH Aachen University, Aachen, Germany; Department of Medicine I, Gastroenterology and Hepatology, Faculty of Medicine and University Hospital Carl Gustav Carus, TUD Dresden University of Technology, Dresden, Germany; Center for Regenerative Therapies Dresden (CRTD), TUD Dresden University of Technology, Dresden, Germany; Else Kroener Fresenius Center for Digital Health, Faculty of Medicine and University Hospital Carl Gustav Carus, TUD Dresden University of Technology, Dresden, Germany; Department of Internal Medicine V—Pneumology, University Hospital Aachen, RWTH Aachen University, Aachen, Germany; Cardiocentro Ticino Institute, Ente Ospedaliero Cantonale (EOC), Lugano, Switzerland; Faculty of Biomedical Sciences, University of Italian Switzerland, Lugano, Switzerland; Department of Vascular Surgery, University Hospital Aachen, RWTH Aachen University, Aachen, Germany; Division of Cardiology, Department of Medicine and the Centre for Cardiovascular Innovation, Vancouver General Hospital, University of British Columbia, Vancouver, British Columbia, Canada; National Disease Registration Service, NHS, 10 South Colonnade, Canary Wharf, London E14 4PU, UK; Center for Molecular Cardiology, University of Zürich, Wagistrasse 12, 8952 Schlieren, Switzerland; Department of Cardiovascular Sciences, University of Leicester, University Road, Leicester LE1 7RH, UK; Department of Clinical Sciences, Karolinska Institute, 171 77 Stockholm, Sweden; Duke University Medical Center; Division of Cardiology, Department of Medicine, Duke University School of Medicine, Durham, North Carolina, USA; Heart Institute (Instituto do Coracao (InCor)), University of Sao Paulo Medical School Hospital, Sao Paulo, Brazil; Duke University Medical Center; Division of Cardiology, Department of Medicine, Duke University School of Medicine, Durham, North Carolina, USA; Division of Cardiology, Research Institute of the McGill University Health Centre, McGill University, Montreal, Quebec, Canada; Department of Internal Medicine I—Cardiology, University Hospital Aachen, RWTH Aachen University, Pauwelsstraße 30, Aachen D-52074, Germany; Department of Cardiovascular Medicine, Tohoku University Graduate School of Medicine, 1-1 Seiryo-machi, Aoba-ku, Sendai, Miyagi 980-8574, Japan; International University of Health and Welfare, Chiba 286-8686, Japan; Department of Cardiovascular Sciences—CUORE, Fondazione Policlinico Universitario A. Gemelli—IRCCS, Rome, Italy; Catholic University of the Sacred Heart, Rome, Italy; Department of Cardiology, Hacettepe University Medical Faculty, Ankara, Türkiye; Centre of Excellence of Cardiovascular Sciences, Gemelli Isola Hospital, Rome, Italy; Center for Molecular Cardiology, University of Zürich, Wagistrasse 12, 8952 Schlieren, Switzerland; Royal Brompton & Harefield Hospitals, Imperial College and King's College, London, UK; Heart and Vascular Institute, Mass General Brigham Hospital, and Harvard Medical School, Boston, MA, USA; Heart and Vascular Institute, Mass General Brigham Hospital, and Harvard Medical School, Boston, MA, USA; Center for Cardiovascular Disease Prevention, Division of Preventive Medicine, Brigham and Women’s Hospital, Boston, MA, USA; Department of Internal Medicine I—Cardiology, University Hospital Aachen, RWTH Aachen University, Pauwelsstraße 30, Aachen D-52074, Germany; Department of Internal Medicine III—Gastroenterology, University Hospital Aachen, RWTH Aachen University, Aachen, Germany; Department of Internal Medicine I—Cardiology, University Hospital Aachen, RWTH Aachen University, Pauwelsstraße 30, Aachen D-52074, Germany

**Keywords:** high-sensitivity C-reactive protein, Cardiovascular risk prediction, Primary prevention, UK Biobank, Risk assessment, Biomarker

## Abstract

**Background and Aims:**

High-sensitivity C-reactive protein (hsCRP) is a marker of inflammation and predicts cardiovascular (CV) risk in individuals without known atherosclerotic CV disease (ASCVD). More information about its clinical relevance will help evaluate the general utility of hsCRP as a routine clinical biomarker to identify patients at residual risk.

**Methods:**

In this population-based study, hsCRP was measured in 448 653 UK Biobank participants without known ASCVD. The association of hsCRP with major adverse cardiovascular events (MACE), CV death and all-cause death was assessed using Cox proportional hazards models.

**Results:**

The cohort had a median age of 57 years, 55.4% were female, and median hsCRP levels were 1.32 mg/L. A repeat hsCRP measurement in 15 967 participants after 4.4 years showed long-term stability. In covariate-adjusted models individuals with hsCRP levels >3 mg/L had a 34% higher risk of MACE, a 61% and 54% increased risk of CV death and all-cause death compared to those with hsCRP <1 mg/L. Subjects with hsCRP levels ≥2 mg/L vs <2 mg/L had a 22% increased risk of MACE, and a 37% and 34% higher risk of CV death and all-cause death. The association of hsCRP with all endpoints was consistent across subgroups. Predictive performance of hsCRP ranked above conventional risk factors. Integration of hsCRP improved SCORE2 and provided a total net reclassification improvement of 14.1% for prediction of MACE.

**Conclusions:**

These data confirm hsCRP as a clinically relevant predictor of CV events in individuals without known ASCVD and support its assessment in primary prevention.

## Structured Graphical Abstract

**Structured Graphical Abstract:**
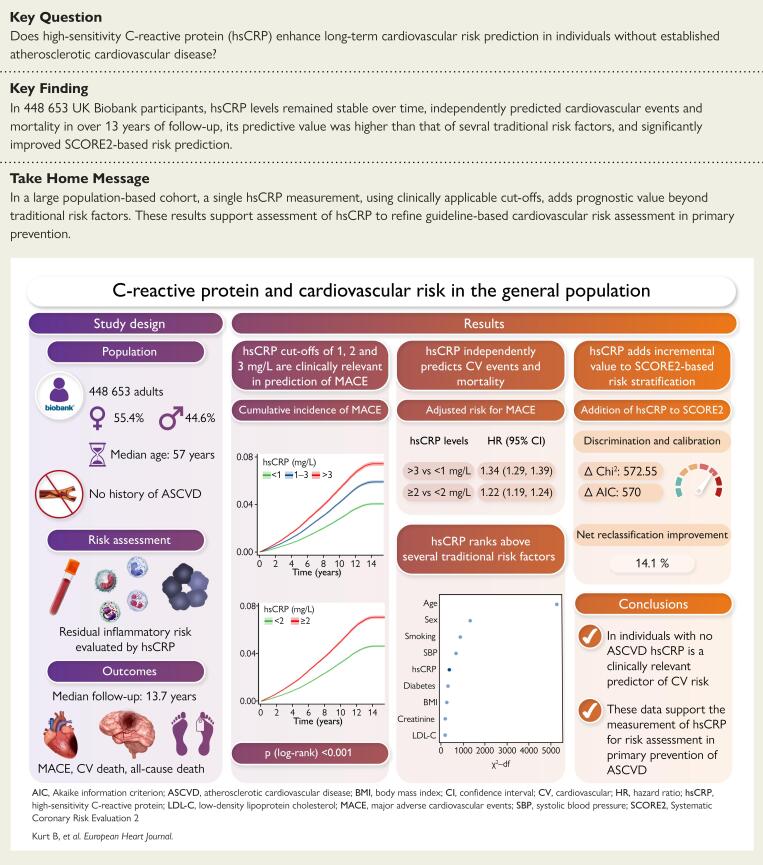
In 448 654 adults without known atherosclerotic cardiovascular disease (ASCVD), high-sensitivity C-reactive protein (hsCRP) independently predicts cardiovascular events and mortality, ranks above traditional risk factors in prediction of outcomes, and adds incremental value to SCORE2-based risk prediction. Routine hsCRP assessment should be considered for inclusion in future guidelines to refine cardiovascular risk assessment and to improve prevention strategies in patients without a history of ASCVD.

**This paper was guest edited by Christian Hamm See the editorial comment for this article ‘Another brick or another wall? High sensitivity C-reactive protein for primary prevention’, by E. Giannitsis *et al*., https://doi.org/10.1093/eurheartj/ehaf1056.**

## Introduction

Despite substantial advances in prevention and treatment strategies over the past decades, cardiovascular (CV) diseases remain the leading cause of morbidity and mortality.^1^ Atherosclerotic CV diseases (ASCVD), including coronary artery disease (CAD), contribute substantially to this global health burden, underscoring the need for more effective risk assessment and management strategies.^2^ ASCVD has multifactorial pathophysiology, driven by modifiable risk factors such as dyslipidemia, hypertension and smoking, alongside non-modifiable characteristics including age, sex and genetic predisposition.^3,4^ While traditional factors are central to prevention strategies, they fail to reflect fully the substantial residual risk observed in many individuals. Growing evidence supports inflammation as a fundamental driver of residual risk, contributing causally to the development of ASCVD and its complications.^5–8^ The identification of individuals with residual inflammatory risk remains a key challenge in preventive strategies, highlighting the need for reliable biomarkers to refine risk stratification and guide targeted interventions. Internationally recognized risk scores, such as the Systematic COronary Risk Evaluation 2 (SCORE2) in Europe, are integral tools in primary prevention and integrate demographic and clinical variables such as age, sex, blood pressure and lipid levels to estimate CV risk.^9–11^ While traditional risk factors are central to prevention strategies, residual CV risk remains inadequately addressed, with inflammation recognized as a fundamental underlying driver.^12,13^

Inflammatory biomarkers, such as high-sensitivity C-reactive protein (hsCRP), have emerged as widely available and important biomarkers for risk stratification and have undergone extensive study for their prognostic value in CV risk prediction.^14–16^ HsCRP helps to gauge residual inflammatory risk, which contributes to the pathogenesis of ASCVD and CV outcomes, even in those with well-controlled conventional risk factors, such as those with cholesterol levels at target.^17–22^ Moreover, recent investigations reinforced the predictive value of hsCRP concentrations in primary prevention.^23,24^ These findings highlight the potential of inflammatory biomarkers to refine risk assessment models and help guide diagnostic and therapeutic decision-making, especially in subjects without established ASCVD.

Regardless of the established role of hsCRP as a biomarker for low-grade systemic inflammation with demonstrated capacity in improving CV risk prediction, its routine clinical use remains an area of debate. Current guideline recommendations vary by region: Canadian and US guidelines endorse the measurement of hsCRP in selected individuals without ASCVD.^25,26^ Although measurement of hsCRP is recommended in secondary prevention, such as within the SMART score—a risk tool of the European Society of Cardiology (ESC) for evaluation of CV events in patients with established ASCVD—it remains significantly underutilized in primary prevention.^27,28^ However, the 2024 ESC Guidelines for chronic coronary syndromes recommended assessing hsCRP in patients with suspected CAD, which reflects accumulating evidence in support of the use of hsCRP for risk stratification.^29^ Despite these recent guideline updates, routine clinical implementation of hsCRP lags behind. As its position in CV risk assessment across different contexts of use within diverse populations remains incompletely defined, data supporting the use of hsCRP in primary prevention continues to engender discussion. Previous studies were limited by small sample sizes, inconsistent integration into established risk models and scarce use of advanced biostatistical methodology. These concerns have contributed to the lack of established guideline recommendations for hsCRP use in primary prevention.

Closing this gap of knowledge requires a comprehensive evaluation in population-based cohorts to assess the clinical utility of hsCRP as a useful biomarker that can achieve predictive capacity comparable to and add information beyond established markers such as low-density lipoprotein cholesterol (LDL-C) and blood pressure in primary prevention.

We therefore conducted a large-scale evaluation of hsCRP in individuals without known ASCVD to assess its independent prognostic value, temporal stability, and ability to improve established risk models. Using contemporary statistical methods, we examined its contribution to discrimination, calibration, and reclassification, with a focus on clinically relevant subgroups and guideline-recommended cut-points.

## Methods

### Study population and clinical data

The UK Biobank is a population-based cohort study comprising over 500 000 participants aged 37–73 years at baseline. Participants were enrolled between 2006 and 2010, during which period demographic data, medical history and lifestyle factors were collected through structured questionnaires and physical examinations, including anthropometric and vital sign measurements, were performed. Biological samples were collected for comprehensive laboratory and biochemical analyses. A subset of 15 967 participants who attended a repeat assessment visit between 2012 and 2013 was included for additional analyses. Participants provided electronic consent for data linkage to medical records and national health registries. Clinical characteristics, baseline medical conditions, medication and longitudinal follow-up outcomes with date of first occurrence were assessed with self-reported questionnaires, hospital admission records, national cancer registries and death registration databases and coded with the International Statistical Classification of Diseases and Related Health Problems, Tenth Revision (ICD-10). Individuals without a known history of ASCVD and age ≥40 years at baseline were included in this study. The primary outcomes were a composite CV endpoint comprising CV death, non-fatal myocardial infarction or non-fatal stroke (major adverse cardiovascular events, MACE) as well as CV death and all-cause death. Further details on ICD-10-code-based definitions are provided in Supplementary data online, *Table S1*.

### Laboratory measurements

The UK Biobank Biomarker Enhancement Project conducted laboratory analyses on samples collected at baseline (2006–10) and during a repeat assessment phase (2012–13). Serum biomarkers of clinical chemistry included total cholesterol, high-density lipoprotein cholesterol (HDL-C), direct LDL-C, creatinine, estimated glomerular filtration rate (eGFR using the 2009 Chronic Kidney Disease Epidemiology Collaboration formula) and glycated haemoglobin (HbA1c). Biomarkers were analysed using enzymatic methods and immunoassays on a Beckman Coulter AU5800 analyzer and the Bio-Rad Variant II Turbo analyzer to determine the relative concentration of HbA1c. Serum measurement of hsCRP was performed using a high-sensitivity immunoturbidimetric assay on a Beckman Coulter AU5800 analyzer with results reported within a validated range of 0.08–80 mg/L. For samples exceeding this range, automatic or manual dilution was performed to bring results within the reportable range. Quality assurance included internal quality controls, external quality assurance schemes and inter-instrument comparison to maintain consistency across analytical platforms. For hsCRP within-laboratory (total) precision is reported with coefficients of variation of 2.31%, 1.70%, and 1.69% at low, medium, and high quality-control levels. Full laboratory information is publicly available on the UK Biobank website.

### Statistical analysis

Continuous variables are expressed as median with interquartile range [Q1, Q3], and categorical variables are shown as absolute and relative frequencies (%). To test for overall differences in continuous variables across groups, one-way analysis of variance (ANOVA) was used. *P*-values refer to the null-hypothesis of no difference between any of the group means. Biomarker levels of hsCRP were plotted in histograms for graphical representation.

To evaluate long-term stability of hsCRP, serial measurements were analysed in a subset of participants who underwent a second biomarker assessment between 2012 and 2013 following their initial baseline evaluation between 2006 and 2010. To visualize the distribution as well as continuous and categorical shifts of hsCRP levels over time, violin plots, flowline (spaghetti) plots showing individual changes and alluvial plots illustrating transitions between hsCRP categories were generated.

Kaplan-Meier survival analyses with log-rank tests were assessed to evaluate time-to-event data for each primary endpoint (MACE, CV death and all-cause death), with hsCRP categorized into groups of <1 mg/L, 1–3 mg/L, and >3 mg/L as well as dichotomized at 2 mg/L. Univariable and multivariable Cox proportional hazards models were calculated to estimate hazard ratios (HRs) with 95% confidence intervals (CIs) for hsCRP in relation to the primary endpoints. Analyses were performed with hsCRP as a continuous variable (interquartile HR) and comparing median levels of hsCRP categories (<1 mg/L, 1–3 mg/L, >3 mg/L; ≥2 mg/L, <2 mg/L), providing relative and absolute risk estimates. Main multivariable-adjusted analyses were performed in a two-step approach with a model including established CV risk factors based on significant associations with baseline hsCRP levels, direct comparators and prior evidence^18,23^ (Model 1) as well as a fully-adjusted model including Model 1 and an extensive set of additional potential confounders of hsCRP levels (Model 2). Further sensitivity analyses were performed with eGFR instead of creatinine for both models (Model 3, Model 4). Partial effect plots were computed for visualization of clinical variables with CV outcomes. Interactions between included variables were analysed and incorporated into models as necessary. Variable importance analyses with corresponding plots illustrated the relative contribution of hsCRP compared to the covariates in the models. Clinically relevant subgroups were analysed to assess their influence on the association between hsCRP and outcomes. Forest plots were used to display adjusted HRs, 95% CIs and *P*-values of interaction for median levels of categorized hsCRP levels. Prognostic relevance of hsCRP in the subgroup of subjects without known standard modifiable CV risk factors (SMuRFs) was additionally assessed in comparison to the full cohort. SMuRFs were defined based on previously published work.^30^ The full definition of ‘SMuRF-less’ criteria applied to our study is provided in the Supplementary data online, *Methods*.

To evaluate the incremental added value of hsCRP, CV risk was quantified using subsumed SCORE2, SCORE2-OP (Older Persons) and SCORE2-Diabetes as the guideline-recommended risk scores for a European cohort^9–11^ and recalculated with hsCRP as an additional covariate. Model fit was investigated by application of likelihood ratio (LR) Chi^2^ tests and the Akaike Information Criterion (AIC). Continuous net reclassification improvement (NRI) was assessed to evaluate the incremental prognostic value of hsCRP when added to the SCORE2 model for prediction of 10-year risk of MACE. All statistical analyses were performed using R software (version 4.5.0). Further information is provided in the Supplementary data online, *Methods*.

## Results

### Baseline characteristics of study participants

Clinical and laboratory baseline characteristics stratified by accepted clinically useful cutpoints into hsCRP concentrations of <1 mg/L, 1–3 mg/L and >3 mg/L are shown in *Table 1*. A total of 448 653 participants with available hsCRP measurements, no known prior ASCVD, and median age of 57 [50, 63] years were included in our study. Median hsCRP levels were 1.32 [0.65, 2.74] mg/L (see Supplementary data online, *Figure S1*). Among all participants, 55.4% were female, median body mass index (BMI) was 26.7 [24.1, 29.8] kg/m^2^, 10.4% were current smokers and 1.3% had type 2 diabetes mellitus. A total of 67 712 (15.1%) of individuals assessed had no known SMuRF at baseline. Further data on ethnic backgrounds available in the UK Biobank is provided in Supplementary data online, *Table S2* and numbers of missing baseline parameters are specified in *Table S3*.

**Table 1:** Baseline characteristics

Characteristics	All participants (*n* = 448 653)	hsCRP <1 mg/L (*n* = 177 007)	hsCRP 1–3 mg/L (*n* = 170 811)	hsCRP >3 mg/L (*n* = 100 835)
Demographics				
Age—years	57 [50, 63]	56 [48, 62]	58 [51, 63]	59 [51, 64]
Female sex—no. (%)	248 763 (55.4)	96 645 (54.6)	91 181 (53.4)	60 937 (60.4)
White—no. (%)	422 929 (94.3)	167 060 (94.4)	161 329 (94.4)	94 540 (93.8)
CV risk factors				
BMI—kg/m^2^	26.7 [24.1, 29.8]	24.9 [22.8, 27.3]	27.4 [24.9, 30.2]	29.4 [26.3, 33.4]
Systolic blood pressure—mmHg	136 [125, 150]	133 [122, 146]	138 [127, 151]	139 [128, 152]
Hypertension—no. (%)	27 045 (6.0)	6598 (3.7)	10 705 (6.3)	9742 (9.7)
Dyslipidemia—no. (%)	7301 (1.6)	2115 (1.2)	3051 (1.8)	2135 (2.1)
Smoker—no. (%)	46 627 (10.4)	14 369 (8.1)	17 845 (10.4)	14 413 (14.3)
Type 2 diabetes mellitus—no. (%)	6038 (1.3)	1544 (0.9)	2301 (1.3)	2193 (2.2)
SMuRFless—no. (%)	67 712 (15.1%)	34 648 (19.6%)	21 770 (12.7%)	11 294 (11.2%)
Laboratory				
Total cholesterol—mg/dL	220.2 [192.3, 249.6]	217.1 [190.5, 245.4]	223.1 [194.6, 252.8]	221.0 [191.8, 251.3]
HDL cholesterol—mg/dL	54.5 [45.7, 65.1]	58.0 [48.6, 68.8]	53.4 [45.1, 63.7]	50.6 [42.9, 60.1]
LDL cholesterol—mg/dL	137.3 [115.7, 160.1]	133.5[113.0, 155.5]	140.1 [118.1, 163.0]	139.8 [117.2, 163.2]
Creatinine—mg/dL	0.79 [0.69, 0.91]	0.79 [0.69, 0.91]	0.80 [0.70, 0.92]	0.78 [0.68, 0.90]
eGFR—mL/min/1,73m^2^	93.1 [83.3, 100.3]	94.1 [85.2, 101.2]	92.3 [82.4, 99.4]	92.4 [81.6, 99.9]
HbA1c—%	5.4 [5.1, 5.6]	5.3 [5.1, 5.5]	5.4 [5.2, 5.6]	5.5 [5.2, 5.7]
hsCRP—mg/L	1.32 [0.65, 2.74]	0.55 [0.36, 0.75]	1.66 [1.28, 2.19]	5.08 [3.79, 8.08]
SCORE2—%	4.1 [2.4, 6.3]	3.4 [2, 5.5]	4.5 [2.7, 6.6]	4.7 [2.9, 6.9]

Clinical and laboratory baseline characteristics stratified into hsCRP levels of <1 mg/L, 1–3 mg/L and >3 mg/L. Data are presented as median [interquartile range] for continuous variables and number (percentage) for categorical variables. To test for overall differences in continuous variables across the three groups, one-way analysis of variance (ANOVA) was used, with *P*-values referring to the null-hypothesis of no difference between any of the group means. All *P*-values were <.001.

BMI, body mass index; CV, cardiovascular; eGFR, estimated glomerular filtration rate; HbA1c, glycated haemoglobin A1c; HDL cholesterol, high-density lipoprotein cholesterol; hsCRP, high-sensitivity C-reactive protein; LDL cholesterol, low-density lipoprotein cholesterol; SCORE2, Systematic COronary Risk Evaluation; SMuRFless, without Standard Modifiable cardiovascular Risk Factors.

### Serial measurements of hsCRP demonstrate long-term stability

In a subset of 15 967 participants with available repeated measurements after a median period of 4.4 [3.7, 4.9] years, hsCRP levels were comparable between baseline (1.16 [0.59, 2.37] mg/L) and follow-up (1.21 [0.61, 2.42] mg/L), indicating long-term stability of hsCRP (see Supplementary data online, *Figure S2*). At follow-up, 71% of those with hsCRP <1 mg/L, 57.1% of those with hsCRP 1–3 mg/L, and 51.3% of those with hsCRP >3 mg/L at baseline remained in the same category (see Supplementary data online, *Figure S2B*).

### hsCRP independently predicts cardiovascular events and mortality

Median follow-up for MACE was 13.7 [12.9, 14.3] years and was observed in 23 624 individuals. CV death occurred in 6176 and all-cause death in 35 983 subjects, with median observation times of 15.2 [14.51, 15.9] years and 15.3 [14.7, 15.9] years, respectively (*Table 2*).

**Table 2:** Cox regression analyses assessing hsCRP and the risk of CV outcomes

Model	Endpoint	*n*	No of events	Interquartile HR hsCRP (95% CI)	*P*-value*
hsCRP	MACE	448 653	23 624	1.43 (1.40, 1.45)	<.001
	CV death		6176	1.71 (1.65, 1.76)	
	All-cause death		35 983	1.56 (1.54, 1.58)	
Model 1	MACE	431 421	22 586	1.22 (1.19, 1.24)	<0.001
	CV death		5860	1.36 (1.31, 1.41)	
	All-cause death		35 299	1.36 (1.34, 1.39)	
Model 2	MACE	349 725	17 922	1.20 (1.18, 1.23)	<0.001
	CV death		4538	1.35 (1.30, 1.41)	
	All-cause death		26 579	1.32 (1.29, 1.34)	

Uni- and multivariable Cox proportional hazards models with interquartile hazard ratios (HR) and 95% confidence intervals (CI) assessing the association of hsCRP with CV outcomes (MACE, CV death and all-cause death) in individuals without known ASCVD.

Model 1: adjusted for age, sex, body mass index, type 2 diabetes mellitus, smoking, systolic blood pressure, LDL-C, creatinine, hsCRP.

Model 2: Model 1 + infectious diseases, inflammatory bowel diseases, autoimmune diseases, malignant diseases, rheumatoid arthritis, psoriasis, atrial fibrillation and flutter, aortic stenosis, heart failure, MET (Summed MET minutes per week for all activity), corticosteroids, immunosuppressants, statins, RAASi and CCB.

ASCVD, atherosclerotic cardiovascular disease; CCB, calcium channel blockers; CV, cardiovascular; eGFR, estimated glomerular filtration rate; hsCRP, high-sensitivity C-reactive protein; LDL-C, low-density lipoprotein cholesterol; MACE, major adverse cardiovascular events, MET, metabolic equivalent task, RAASi, renin-angiotensin-aldosterone system inhibitors.

^*^P-values refer to the statistical significance of the reported hazard ratios for hsCRP levels in relation to each endpoint.

**Figure 1:**
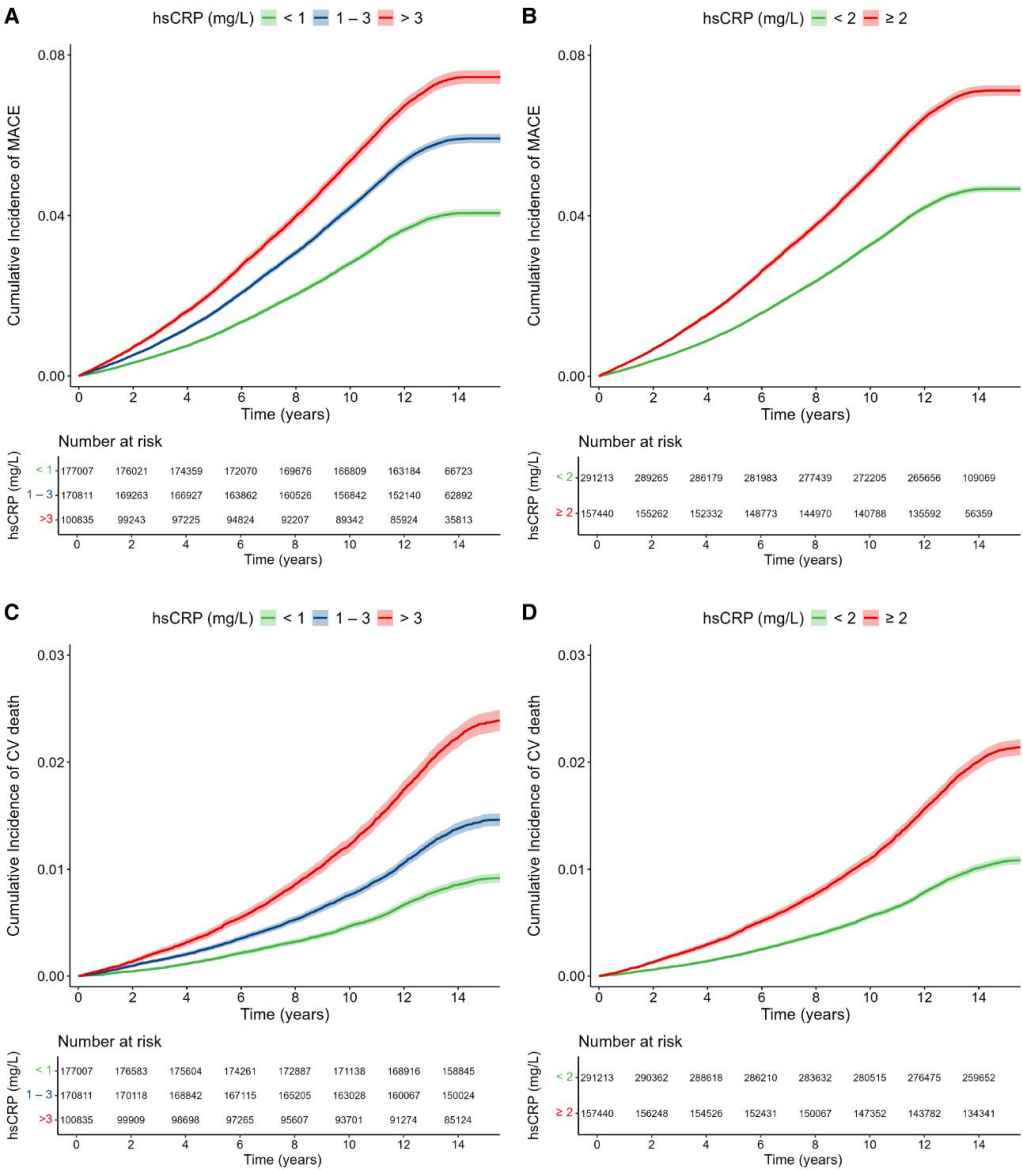
Association of hsCRP with MACE and CV death. Kaplan–Meier curves illustrating the cumulative incidence of CV events stratified by hsCRP levels. The number at risk is displayed below the x-axis and shaded areas represent 95% confidence intervals. (*A*) Incidence of MACE comparing levels of hsCRP <1 mg/L vs. 1–3 mg/L vs. >3 mg/L. (*B*) Incidence of MACE comparing levels of hsCRP <2 mg/L vs ≥2 mg/L. (*C*) Incidence of CV death comparing levels of hsCRP <1 mg/L vs 1–3 mg/L vs >3 mg/L. (*D*) Incidence of CV death comparing levels of hsCRP <2 mg/L vs ≥2 mg/L. All log-rank *P*-values <.001. hsCRP, high-sensitivity C-reactive protein; MACE, major adverse cardiovascular events; CV, cardiovascular

Stratification of hsCRP levels into categories of <1 mg/L, 1–3 mg/L, and >3 mg/L showed a stepwise increase in MACE incidence, with the highest event rates observed in individuals with hsCRP levels >3 mg/L (*Figure 1A*; log-rank *P* < .001). Higher occurrence of MACE was also observed in individuals with hsCRP levels ≥2 mg/L compared to those with hsCRP <2 mg/L (*Figure 1B*; log-rank *P* < .001), another commonly used cut-off point. Similar results were observed for CV death (*Figure 1C* and *D*) and all-cause death (see Supplementary data online, *Figure S3*; all log-rank *P* < .001).

In univariable Cox regression analyses, higher hsCRP levels were significantly associated with increased risk for all endpoints (*Table 2*). For MACE, the interquartile HR was 1.43 (95% CI: 1.40, 1.45, *P* < .001). In a base multivariable model (Model 1) hsCRP remained independently associated with MACE after adjusting for the established CV risk factors age, sex, BMI, diabetes, smoking, systolic blood pressure, LDL-C and creatinine (Model 1: adjusted HR: 1.22; 95% CI: 1.19, 1.24; *P* < .001; *Table 2*). Comparable results were observed for CV death and all-cause death (*Table 2*). Modelling for continuous covariates, including log-transformation and spline functions, is shown in Supplementary data online, *Figure S4*. The fully-adjusted main model (Model 2; *Table 2*) additionally included an extensive set of confounders potentially affecting hsCRP levels including acute and chronic infectious, malignant, autoimmune and inflammatory bowel diseases, other CV comorbidities which are not part of our ASCVD definition (atrial fibrillation and atrial flutter, aortic stenosis and heart failure), metabolic equivalents and use of corticosteroids, immunosuppressants, statins, calcium channel blockers (CCB) and renin-angiotensin-aldosterone system inhibitors (RAASi). This confounder-analysis showed that hsCRP remained a strong predictor of MACE (Model 2: adjusted HR: 1.20; 95% CI: 1.18, 1.23; *P* < .001), CV death (Model 2: adjusted HR: 1.35; 95% CI: 1.30, 1.41; *P* < .001) and all-cause death (Model 2: adjusted HR: 1.32; 95% CI: 1.29, 1.34; *P* < .001) after multivariable adjustment (*Table 2*).

**Figure 2:**
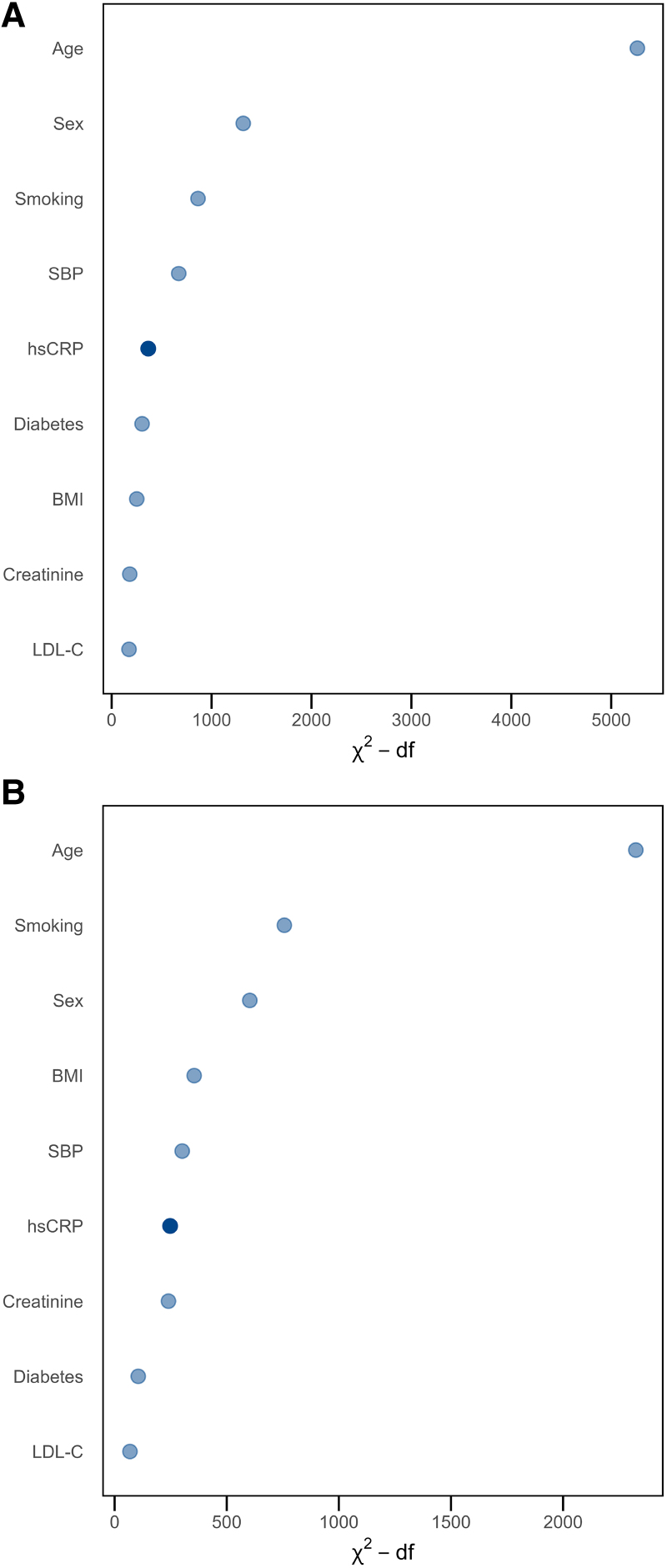
Variable importance of hsCRP in risk prediction of MACE and CV death (model 1). Variable importance plots from multivariable Cox regression models assessing the individual contribution of each variable to overall model fit and risk prediction after adjustment. Variables are derived from Model 1 and include hsCRP (mg/L), age (years), sex, BMI (kg/m^2^), diabetes, smoking, systolic blood pressure (mmHg), LDL-C (mg/dL) and creatinine (mg/dL). The likelihood ratio (LR) Chi^2^ statistic minus the degrees of freedom (Chi^2^—df) is plotted for each model variable. (*A*) Variable importance for the prediction of MACE. (*B*) Variable importance for the prediction of CV death. BMI, body mass index; CV, cardiovascular; hsCRP, high-sensitivity C-reactive protein; LDL-C, low-density lipoprotein cholesterol; MACE, major adverse cardiovascular events; SBP, systolic blood pressure

Variable importance analyses with head-to-head comparison of predictive performance between hsCRP and established CV risk factors ranked hsCRP above traditional risk markers such as LDL-C for predicting MACE, CV death and all-cause death (*Figure 2*; Supplementary data online, *Figure S5*).

To evaluate the prognostic capacity of clinically established hsCRP cut-off values, we performed additional analyses within the fully-adjusted main model (Model 2) with relative and absolute risk estimates across the observation period, provided in *Table 3*. Individuals with hsCRP levels >3 mg/L showed a 34% higher risk of MACE (adjusted HR 1.34; 95% CI: 1.29, 1.39; *P* < .001) compared to those with hsCRP <1 mg/L, translating to absolute risks of 2.63% vs 1.97%. Moreover we observed a 61% higher risk of CV death (adjusted HR 1.61; 95% CI: 1.50, 1.72; *P* < .001) and a 54% higher risk of all-cause death (adjusted HR 1.54; 95% CI: 1.50, 1.58; *P* < .001) in individuals with hsCRP >3 mg/L vs <1 mg/L. Further risk assessment demonstrated that hsCRP levels ≥2 mg/L vs <2 mg/L were associated with a 22% increased relative risk of MACE (adjusted HR: 1.22; 95% CI: 1.19, 1.24; *P* < .001), corresponding to absolute risks of 2.52% vs 2.08%. When compared to hsCRP levels <2 mg/L, subjects with hsCRP levels ≥2 mg/L had a 37% higher risk of CV death (adjusted HR: 1.37; 95% CI: 1.31, 1.44; *P* < .001) and 34% for all-cause death (adjusted HR: 1.34; 95% CI: 1.31, 1.36; *P* < .001).

**Table 3:** Relative and absolute risk estimates for the association of hsCRP with CV outcomes in 349 725 individuals (Model 2)

Endpoint	hsCRP group (mg/L)	*n*	No of events	Crude HR^a^ (95% CI)	Adj. HR^a,b^ (95% CI)	AR^c^ (%) (95% CI)
MACE	<1	142 554	5458	Ref.	Ref.	1.97 (1.89, 2.06)
	1–3	132 859	7335	1.32 (1.30, 1.34)	1.16 (1.14, 1.18)	2.28 (2.19, 2.38)
	>3	74 312	5129	1.74 (1.69, 1.80)	1.34 (1.29, 1.39)	2.63 (2.51, 2.75)
	<2	232 143	10 171	Ref.	Ref.	2.08 (1.99, 2.17)
	≥2	117 582	7751	1.45 (1.42, 1.48)	1.22 (1.19, 1.24)	2.52 (2.41, 2.63)
CV death	<1	142 554	1172	Ref.	Ref.	0.24 (0.22, 0.26)
	1–3	132 859	1789	1.52 (1.47, 1.56)	1.27 (1.23, 1.31)	0.30 (0.28, 0.33)
	>3	74 312	1577	2.30 (2.17, 2.44)	1.61 (1.50, 1.72)	0.39 (0.35, 0.42)
	<2	232 143	2288	Ref.	Ref.	0.26 (0.24, 0.29)
	≥2	117 582	2250	1.75 (1.68, 1.82)	1.37 (1.31, 1.44)	0.36 (0.33, 0.39)
All-cause death	<1	142 554	7766	Ref.	Ref.	2.31 (2.23, 2.39)
	1–3	132 859	10 259	1.41 (1.39, 1.43)	1.24 (1.22, 1.26)	2.86 (2.76, 2.95)
	>3	74 312	8554	1.99 (1.94, 2.04)	1.54 (1.50, 1.58)	3.54 (3.41, 3.66)
	<2	232 143	14 222	Ref.	Ref.	2.50 (2.41, 2.58)
	≥2	117 582	12 357	1.59 (1.56, 1.61)	1.34 (1.31, 1.36)	3.32 (3.20, 3.43)

Uni- and multivariable Cox proportional hazards models assessing the association of hsCRP with CV outcomes (MACE, CV death and all-cause death) during the observation period in individuals without known ASCVD. Hazard ratios (HR) and absolute risks (AR) with 95% confidence intervals (CI) are provided for hsCRP levels categorized into groups of <1 mg/L, 1–3 mg/L, and >3 mg/L as well as dichotomized at 2 mg/L derived from established cut-offs from guidelines and previous studies. Uni- and multivariable models were performed within the multivariable model cohort. The <1 mg/L and <2 mg/L groups serve as reference for respective comparisons.

AR, absolute risk; ASCVD, atherosclerotic cardiovascular disease; CCB, calcium channel blockers; CV, cardiovascular; eGFR, estimated glomerular filtration rate; hsCRP, high-sensitivity C-reactive protein; LDL-C, low-density lipoprotein cholesterol; MACE, major adverse cardiovascular events; MET, metabolic equivalent task, RAASi, renin-angiotensin-aldosterone system inhibitors; Ref., reference value.

^a^All *P*-values were <.001.

^b^Multivariable model (Model 2) adjusted for age, sex, body mass index, type 2 diabetes mellitus, smoking, systolic blood pressure, LDL-C, creatinine, hsCRP, infectious diseases, inflammatory bowel diseases, autoimmune diseases, malignant diseases, rheumatoid arthritis, psoriasis, atrial fibrillation and flutter, aortic stenosis, heart failure, MET (summed MET minutes per week for all activity), corticosteroids, immunosuppressants, statins, RAASi and CCB.

^c^Absolute risks were computed using the multivariable Cox regression models, with the risks assessed over the entire follow-up period.

Assessing the hsCRP categories of <1 mg/L, 1–3 mg/L, and >3 mg/L or dichotomized at 2 mg/L within the base multivariable model (Model 1) demonstrated consistent associations with MACE, CV death and all-cause death in 431 421 individuals (see Supplementary data online, *Table S4*).

Moreover, in sensitivity analyses predictive performance and variable importance of hsCRP were not attenuated by use of eGFR instead of creatinine (see Supplementary data online, *Table S5*–*S7*; Supplementary data online, *Figure S6* and *S7*).

### The predictive value of hsCRP remains stable across clinical subgroups and in SMuRF-less subjects

The association between hsCRP and MACE, CV death and all-cause death remained significant in the multivariable model for predefined clinical subgroups defined by age, sex, BMI, type 2 diabetes mellitus, hypertension, smoking status, eGFR, LDL-C and dyslipidemia (see Supplementary data online, *Figure S8*–*S10*). Although interaction terms in the multivariable model pointed to possible heterogeneity in effect size across subgroups, the association between higher hsCRP levels and increased risk remained consistent, with all hazard ratios and 95% CIs exceeding 1.

Moreover, in subgroup analyses performed in SMuRF-less subjects, cumulative incidence showed a stepwise increase across hsCRP categories of <1 mg/L, 1–3 mg/L, and >3 mg/L and for subjects with hsCRP levels >2 mg/L compared to those <2 mg/L for MACE (see Supplementary data online, *Figure S11A* and *B*), CV death (see Supplementary data online, *Figure S11C* and *D*), and all-cause death (see Supplementary data online, *Figure S11E* and *F*) in the absence of traditional SMuRFs (all log-rank *P* < .001).

### hsCRP provides incremental value for SCORE2-based risk stratification

The addition of hsCRP improved the predictive performance and model fit of SCORE2 for 10-year risk of MACE as indicated by a significant increase in LR Chi^2^ (SCORE2: Chi^2^ = 5358.30; SCORE2 + hsCRP: Chi^2^ = 5930.85; Δ Chi^2^ = 572.55; *P* < .001) and a decrease in AIC (Δ AIC = 570) (*Table 4*). Reclassification analyses confirmed the enhancement in risk prediction when hsCRP was added to SCORE2, with an NRI for MACE of 2.4% and a non-event NRI of 11.7%, resulting in a total continuous net reclassification improvement of 14.1% (*Table 4*).

**Table 4:** Model performance of hsCRP in addition to SCORE2

Model	*n*	No of events	Chi^2^	Δ Chi^2^	*P*-value*	AIC	Δ AIC	NRI (MACE)	NRI (event-free)	Total NRI
SCORE2	396 363	15 092	5358.30	572.55	<.001	382 499	570	2.4%	11.7%	14.1%
SCORE2 + hsCRP			5930.85			381 929				

Multivariable Cox proportional hazards models evaluating the addition of hsCRP to the subsumed SCORE2 for prediction of 10-year MACE risk. The model performance was assessed without and with hsCRP as an additional variable. Model performance was evaluated using changes in LR Chi^2^ statistics (Δ Chi^2^) and Akaike Information Criterion (AIC). Expected continuous net reclassification percentages for individuals who experienced MACE and those who remained event-free during the observation period, as well as the resulting total continuous NRI are shown.

SCORE 2, Systematic COronary Risk Evaluation 2; hsCRP, high-sensitivity C-reactive protein; AIC, Akaike Information Criterion; NRI, net reclassification improvement.

^*^P-value indicates the statistical significance of model improvement after adding hsCRP to SCORE2, based on the LR Chi^2^ test.

## Discussion

This study likely represents the largest analysis to date with long-term follow-up assessing the predictive performance and clinical relevance of hsCRP for CV risk in comparison with other biomarkers in a single cohort of low-risk individuals without known ASCVD. While the routine integration of hsCRP into CV risk assessment remains debated, the results presented here affirm hsCRP as a strong and independent predictor of CV events in primary prevention (*Structured Graphical Abstract*).

Current Canadian and US guidelines recommend hsCRP measurement in individuals without ASCVD to improve risk classification, whereas prior European guidelines do not recommend routine hsCRP measurement in primary prevention.^25,26,28^ Our findings confirm that hsCRP independently enhances CV risk stratification, and the predictive performance of hsCRP was comparable to or greater than traditional risk factors such as systolic blood pressure or LDL-C.^17–19,23^

Some previous studies reported only modest incremental predictive value of hsCRP in primary prevention.^18,31^ However, findings were limited by various sample sizes and a predominant reliance on C-index as a measure of model performance. While the C-index is widely used to assess discrimination, it likely underestimates the incremental value of novel biomarkers, especially concerning models where traditional risk factors already show strong discrimination, restricting the potential of novel predictors to add incremental value.^12,13,32,33^ To address these limitations and enable a more nuanced assessment of biomarker utility, our study applied contemporary statistical methodology beyond application of the C-index, such as LR Chi^2^ and AIC, providing a validated approach for assessing incremental predictive value, model fit, discrimination and calibration. Beyond demonstrating independent associations with outcomes, this study shows that hsCRP substantially enhances risk prediction when integrated into SCORE2, as shown by improvements in LR Chi^2^ and AIC. Complementing model fit statistics, we examined the NRI, quantifying the clinical utility and practicability of incorporating hsCRP into SCORE2. The NRI quantifies how much a new biomarker improves the correct classification of individuals into clinically relevant risk categories compared to a baseline model.^34^ Integration of hsCRP into SCORE2 resulted in a continuous net reclassification improvement of 14.1% among individuals with and without CV events. This improvement was mainly driven by more accurate assignment of lower predicted risk in event-free individuals, reflecting enhanced specificity, whereas gains in sensitivity were more modest. These results further highlight the potential of hsCRP for enhancing individualized risk prediction.

Notably, hsCRP remained associated with CV risk across all evaluated subgroups, including low-risk populations such as those with a BMI <25 kg/m^2^, LDL-C < 100 mg/dL or those without hypertension, type 2 diabetes or a history of smoking, while CV risk factors in general were infrequent. Interestingly, even in patients without SMuRFs, high hsCRP levels were associated with an increased incidence of MACE, CV death and all-cause death (see Supplementary data online, *Figure S11*). These findings support the clinical relevance of low-grade systemic inflammation even among those without apparent traditional risk factors.

Moreover, participants were not selected based on baseline hsCRP levels or pre-existing conditions (except known ASCVD), enabling comprehensive risk assessment across the general population. This approach was chosen to reduce selection bias and enhance generalizability. Providing further real-world evidence of large-scale hsCRP distributions in primary prevention cohorts, our analyses showed that presence of markedly elevated hsCRP levels was very low (median hsCRP: 1.32 [.65, 2.74] mg/L) and the majority of the cohort remained within the range linked to low-grade systemic inflammation (<10 mg/L; Supplementary data online, *Figure S1*). Importantly, even after extensive multivariable adjustment for potential confounders which are often debated as factors complicating assessment of hsCRP in clinical routine, prediction of MACE, CV death and all-cause death was not attenuated. Notably, due to the cohort size and number of events, this extent of adjustment was feasible without compromising model stability.

Another key finding of our study was that serial measurements within the UK Biobank confirmed that hsCRP levels remained stable over time. Specifically, 71% of individuals with hsCRP levels <1 mg/L and over half of those in the 1–3 mg/L and >3 mg/L groups retained their baseline category, addressing concerns regarding its biological variability and highlighting its reliability as a clinically useful biomarker. Nonetheless, variability over time is a bias towards the null suggesting that, if anything, we have underestimated the true biologic risk associated with inflammation as detected by hsCRP. These results concur with the stability of hsCRP during repeated measurements over years in the placebo group of JUPITER.^35^

In summary, these findings support hsCRP as a biomarker for broader clinical implementation, demonstrating that a single measurement predicts long-term CV outcomes and provides incremental value in CV risk prediction.

The U.S. Food and Drug Administration (FDA) and The Biomarkers, EndpointS and other Tools (BEST) glossary outline criteria for the clinical implementation of biomarkers.^36^ Beyond its role as a risk- and prognostic biomarker, hsCRP could serve to guide anti-inflammatory therapy. Large clinical trials, including JUPITER and CANTOS have demonstrated that targeting residual inflammatory risk defined as hsCRP levels ≥2 mg/L significantly reduces CV events^37,38^ and multiple ongoing global trials are assessing interleukin-6 inhibition among individuals with elevated hsCRP.^39^ Given universal availability, low cost, easy interpretability of hsCRP, future studies should explore whether hsCRP measurement can identify patients most likely to benefit from anti-inflammatory therapies.

Several limitations of our study must be acknowledged. First, this study of participants aged ≥40 years was conducted within a single cohort, which may limit generalizability to different baseline risk profiles or geographic diversity with other than predominantly European and Caucasian ancestry, as 94.3% of the subjects assessed where White. Second, diagnoses were either self-reported or based on ICD codes, which introduce a degree of inaccuracy. Moreover, participants were without known ASCVD. Unrecognized subclinical atherosclerosis at baseline may have led to misclassification and could partly account for the prognostic signal attributed to hsCRP. Furthermore, no comprehensive exclusion criteria were applied to reduce selection bias. This strategy enabled assessment of systemic inflammation as a predictor of CV risk, independent of its underlying cause, and ensured that no subgroup was disproportionately underrepresented. However, since participation in the UK Biobank required voluntary attendance at assessment centres, a healthy cohort bias cannot be excluded, limiting external validity of our results. Moreover, as medical characteristics of the participants are limited, influences after the inclusion and blood draw are often unknown and residual confounding cannot be excluded. Nevertheless, to address this limitation, we adjusted for various chronic comorbidities present at baseline and observed no relevant attenuation of prognostic capacity.

Methodologically in reclassification analyses, it is important to note that the overall NRI combines event and non-event components calculated from different population subgroups and the much larger non-event group may exert disproportionate influence, which requires cautious interpretation of the overall NRI.

Finally, although the statistical associations observed in our study are strong and consistent, the clinical utility remains to be established as absolute benefits could appear modest and generalizability may be limited.

Taken together, this analysis of 448 653 individuals without known ASCVD from the population-based UK Biobank demonstrates a strong and independent association of hsCRP and long-term CV outcomes. We observed stable hsCRP levels across serial measurements and consistent prognostic value for MACE, CV death and all-cause death across all evaluated subgroups. Finally, the integration of hsCRP into SCORE2 improved model performance for prediction of MACE. Routine hsCRP assessment should be considered for inclusion in future guidelines to refine CV risk assessment and to improve prevention strategies in patients without history of ASCVD.

## Supplementary Material

ehaf937_Supplementary_Data

## Declarations

### Data Availability

Individual-level data can be accessed through the UK Biobank upon application and approval (www.ukbiobank.ac.uk). Summary statistics generated in this study are available from the corresponding author upon reasonable request.
