# Supplementary material for: C-reactive protein and cardiovascular risk in the general population
Source: Eur Heart J. 2025 Dec 11;47(15):1799–810. doi: 10.1093/eurheartj/ehaf937 (PMC13101082; doi:10.1093/eurheartj/ehaf937)
Supplement: ehaf937_Supplementary_Data [file ehaf937_Supplementary_Data.pdf]

Supplementary Appendix

Supplement to: Kurt B, Reugels M, Schneider KM, Spiesshoefer J, Milzi A, Gombert A, Fordyce CB, Wenzl FA, Pagidipati NJ, Rocha V, Fudim M, Sharma A, Lehrke M, Shimokawa H, Liuzzo G, Tokgozoglu L, Crea F, Lüscher TF, Libby P, Ridker PM, Marx N, Schneider CV, Kahles F. C-reactive protein and cardiovascular risk in the general population.

This appendix has been provided by the authors to give readers additional information about the work.

Table of contents

Supplemental methods 3

Table S1. Definitions of ICD-10 code based variables and outcomes 5

Table S2. Baseline ethnicity across hsCRP categories 8

Table S3. Missing data for baseline variables 9

Table S4. Relative and absolute risk estimates for the association of hsCRP with CV outcomes in 431 421 individuals (Model 1) 10

Table S5. Cox regression analyses assessing hsCRP and the risk of CV outcomes (Model 3 and Model 4) 11

Table S6. Multivariable relative and absolute risk estimates for the association of hsCRP with CV outcomes (Model 3) 12

Table S7. Multivariable relative and absolute risk estimates for the association of hsCRP with CV outcomes (Model 4) 13

Table S8. Baseline medication across hsCRP categories 14

Figure S1. Distribution of hsCRP levels at baseline 15

Figure S2. Long-term stability in serial hsCRP measurements 17

Figure S3. Association of hsCRP with all-cause death 19

Figure S4. Partial effect plots for associations of clinical variables with CV outcomes (Model 1) 20

Figure S5. Variable importance of hsCRP in risk prediction of all-cause death (Model 1) 22

Figure S6. Partial effect plots for associations of clinical variables with CV outcomes (Model 3) 23

Figure S7. Variable importance of hsCRP in risk prediction of MACE, CV death and all-cause death (Model 3) 25

Figure S8. Association of hsCRP levels with MACE across clinical subgroups 27

Figure S9. Association of hsCRP levels with CV death across clinical subgroups 30

Figure S10. Association of hsCRP levels with all-cause death across clinical subgroups 33

Figure S11. Association of hsCRP with CV outcomes in the full cohort and in individuals without standard modifiable CV risk factors (SMuRF) 36

Supplementary references 39

Supplemental methods

The analyses provided in this manuscript aim to provide deeper insights to the clinical applicability of hsCRP in a single primary prevention cohort with applying contemporary statistical methodology. In our analyses, we identified the subset of individuals without a prior history of ASCVD. ASCVD was defined according to prior European guidelines for primary and secondary prevention (1-3). Three outcomes were investigated: (i) a composite endpoint of cardiovascular (CV) death, non-fatal myocardial infarction, and non-fatal stroke (MACE), (ii) CV death, and (iii) all-cause mortality. CV death was defined as death attributable to cardiovascular disease (ICD codes I00–I99). For diagnoses based on ICD codes primary/first level ICD codes have been utilized. The evaluation of hsCRP as a risk biomarker across as context of use was based on the criteria outlined in the FDA and BEST (Biomarkers, EndpointS, and other Tools) glossary (4).

Categories of hsCRP were analyzed in categories of <1 mg/L, 1–3 mg/L, and >3 mg/L as well as dichotomized at 2 mg/L derived from established cut-points from guidelines and previous studies (5-7) to provide a granular assessment of clinically applicable changes of categories. For outcome analyses, median follow-up time was computed by the reverse Kaplan-Meier method for fatal and non-fatal events. Univariable and multivariable Cox proportional hazards models were used to assess the association between hsCRP and the clinical endpoints. Continuous variables were log-transformed if necessary and modeled using restricted cubic splines with four knots to allow for non-linear relationships, where appropriate. Next to relative risk estimates, absolute risks with 95% CIs for hsCRP categories of <1 mg/L, 1–3 mg/L and >3 mg/L as well as below and above or equal to 2 mg/L are provided using multivariable Cox regression models with risks assessed over the entire follow-up period as recently suggested and demonstrated (8, 9). Absolute risks were calculated based on the population included in multivariable Cox regression models, using the survival probabilities estimated by the model.

To evaluate whether the association between hsCRP and the outcomes (MACE, CV mortality, all-cause mortality) varies across clinically relevant subgroups, interaction terms between hsCRP and baseline characteristics were included simultaneously in the multivariable Cox model. These characteristics include sex, type 2 diabetes mellitus, hypertension, smoking status, dyslipidemia and groups of age, BMI, estimated glomerular filtration rate, and LDL cholesterol. The full model includes all main effects and all hsCRP-by-subgroup interaction terms. Group-specific hazard ratios for hsCRP category change were derived from this interaction model using marginal effects based on adjusted model predictions. In the main multivariable models, creatinine was assessed instead of eGFR. Additional sensitivity analyses including eGFR instead of creatinine (model 3 and model 4) were performed to exclude potential differences in effects, with age and sex used as independent covariates. Hazard ratios and 95% confidence intervals were estimated while conditioning on the median values of all other covariates. The statistical significance of interaction terms was assessed using Wald tests.

Subjects without known standard modifiable risk factors (SMuRF) were entitled as "SMuRF-less" and defined by the following criteria: systolic blood pressure <140 mmHg, diastolic blood pressure <90 mmHg, HbA1c <6.5%, glucose <7.0 mmol/L, total cholesterol ≤5.5 mmol/L, LDL cholesterol ≤3.5 mmol/L, no current tobacco smoking, and no ICD-10 diagnosis codes for type 2 diabetes, type 1 diabetes, dyslipidemia, or hypertension. As the original definition of SMuRF is based on different units than those used in our manuscript, we applied the original thresholds and units for this analysis to ensure consistency with prior definitions.

Model fit was evaluated using goodness-of-fit measures including the Akaike Information Criterion (AIC) and likelihood ratio (LR) tests (Chi^2) and their respective changes, as well as changes in LR Chi^2 (Δ Chi^2) and the AIC (Δ AIC), with an increase in LR Chi^2 statistics and decrease in AIC indicating improvement in predictive capacity. This approach was followed since the LR Chi^2 represents a statistically highly efficient approach for assessing whether a biomarker enhances model performance in the context of nested comparisons, while the Akaike Information Criterion also adjusts for model complexity (10). Previous literature has described the LR Chi^2 as a measure sufficient on its own to evaluate the added predictive value of novel biomarkers (11, 12). Moreover, previous studies suggested

that an AIC decrease of >10 is generally considered as a strong indicator of model improvement (13, 14), which was quantified and comparatively assessed in our study. Proportional hazards assumption was assessed by visual inspection of scaled Schoenfeld residuals. Analyses incorporating the SCORE2, SCORE2-Diabetes, and SCORE2-Older Persons (SCORE2-OP) risk models were conducted as complete-case analyses. The scores were calculated for each participant based on demographic and clinical variables (age, sex, systolic blood pressure, cholesterol levels, HbA1c, eGFR and smoking status and region (United Kingdom)) and the individual score model suitable for each subject has been applied. Estimated risk was modeled providing a 10-year risk estimate for fatal and non-fatal CV events (MACE) and subsumed. The respective models were applied to individuals according to their corresponding baseline parameters. Moreover, continuous net reclassification improvement was assessed for the subsumed SCORE2 (SCORE2, SCORE2-OP, and SCORE2-Diabetes) by calculating the proportion of individuals correctly reclassified, with upward reclassification for those who experienced MACE and downward reclassification for those who remained event-free.

Variable	ICD-10 Codes
**ASCVD**	G45.0, G45.1, G45.2, G45.3, G45.4, G45.8, G45.9, H34.0, H34.1, H34.2, H34.8, H34.9, I21.0, I21.1, I21.2, I21.3, I21.4, I21.9, I22.0, I22.1, I22.8, I22.9, I23.0, I23.1, I23.2, I23.3, I23.4, I23.5, I23.6, I23.8, I24.0, I24.1, I24.8, I24.9, I25.0, I25.1, I25.2, I25.3, I25.4, I25.5, I25.6, I25.8, I25.9, I63.0, I63.1, I63.2, I63.3, I63.4, I63.5, I63.6, I63.8, I63.9, I64.-, I65.0, I65.1, I65.2, I65.3, I65.8, I65.9, I66.0, I66.1, I66.2, I66.3, I66.4, I66.8, I66.9, I67.2, I67.9, I69.3, I69.4, I69.8, I70.0, I70.00, I70.01, I70.1, I70.10, I70.11, I70.2, I70.20, I70.21, I70.8, I70.80, I70.81, I70.9, I70.90, I70.91, I71.0, I71.1, I71.2, I71.3, I71.4, I71.5, I71.6, I71.8, I71.9, K55.1, Z95.1, Z95.5
**Atrial fibrillation/-flutter**	I48.-
**Heart failure**	I11.0, I13.0, I13.2, I50.0, I50.1, I50.9
**Aortic stenosis**	I35.0
**Hypertension**	I10.-, I11.-, I11.0, I11.9, I12.-, I12.0, I12.9, I13.-, I13.0, I13.1, I13.2, I13.9, I15.-, I15.0, I15.1, I15.2, I15.8, I15.9
**Dyslipidemia**	E78.0, E78.1, E78.2, E78.3, E78.4, E78.5, E78.8, E78.9
**Type 2 diabetes mellitus**	E11.-
**Malignant diseases**	C00 - C97
**Inflammatory bowel diseases**	K50.-, K51.-
**Autoimmune diseases**	D51.0, D59.0, D59.1, D69.3, E05.0, E05.9, E06.3, E06.5, E10.-, E10.0, E10.1, E10.2, E10.3, E10.4, E10.5, E10.6, E10.7, E10.8, E10.9, G35.-, G36.0, G61.0, G70.0, H22.0, H22.1, K73.2, K74.3, K75.4, K83.0, K90.0, L10.0, L12.0, L13.0, L63.9, L80.-, L90.0, M30.1, M31.3, M31.7, M32.1, M32.8, M32.9, M32.90, M33.-, M33.0, M33.1, M33.2, M33.9, M34.-, M34.0, M34.1, M34.2, M34.8, M34.9, M35.-, M35.0, M35.1, M35.2, M35.3, M35.4, M35.5, M35.6, M35.7, M35.8, M35.9, M35.99, N02.3, N02.8
**Rheumatoid arthritis**	M05.-, M06.-
**Psoriasis**	L40.-
**Infectious diseases**	A00.0, A00.1, A00.9, A01.0, A01.1, A01.2, A01.3, A01.4, A02.0, A02.1, A02.2, A02.8, A02.9, A03.0, A03.1, A03.2, A03.3, A03.8, A03.9, A04.0, A04.1, A04.2, A04.3, A04.4, A04.5, A04.6, A04.7, A04.8, A04.9, A06.2, A06.4, A06.5, A06.6, A07.0, A07.8, A07.9, A08.0, A08.1, A08.2, A08.3, A08.4, A09.9, A15.0, A15.1, A15.2, A15.3, A15.4, A15.5, A15.6, A15.7, A15.8, A15.9, A16.0, A16.1, A16.2, A16.3, A16.4, A16.5, A16.7, A16.8, A16.9, A17.0, A17.1, A17.8, A17.9, A18.0, A18.1, A18.2, A18.3, A18.4, A18.5, A18.6, A18.7, A18.8, A19.0, A19.1, A19.2, A19.8, A19.9, A20.0, A20.1, A20.2, A20.3, A20.7, A20.8, A20.9, A21.0, A21.1, A21.2, A21.3, A21.7, A21.8, A21.9, A22.0, A22.1, A22.2, A22.7, A22.8, A22.9, A23.0, A23.1, A23.2, A23.3, A23.8, A23.9, A24.0, A24.1, A24.2, A24.3, A24.4, A25.0, A25.9, A26.7, A26.8, A26.9, A27.0, A27.8, A27.9, A28.8, A28.9, A30.0, A30.1, A30.2, A30.3, A30.4, A30.5, A30.8, A30.9, A31.0, A31.1, A31.8, A31.9, A32.0, A32.1, A32.7, A32.8, A32.9, A36.0, A36.1, A36.2, A36.3, A36.8, A36.9, A37.0, A37.1, A37.8, A37.9, A39.0, A39.2, A39.3, A39.4, A39.5, A39.8, A39.9, A40.0, A40.1, A40.2, A40.3, A40.8, A40.9, A41.0, A41.1, A41.2, A41.3, A41.4, A41.5, A41.8, A41.9, A42.0, A42.1, A42.2, A42.7, A42.8, A42.9, A48.0, A48.8, A49.0, A49.1, A49.2, A49.3, A49.8, A49.9, A54.0, A54.2, A54.3, A54.4, A54.5, A54.6, A54.8, A54.9, A56.4, A66.0, A66.1, A66.2, A66.3, A66.4, A66.6, A66.7, A66.8, A66.9, A67.0, A67.1, A67.2, A67.3, A67.9, A68.0, A68.1, A68.9, A69.1, A71.0, A71.1, A71.9, A74.0, A74.8, A74.9, A75.0, A75.1, A75.2, A75.3, A75.9, A77.0, A79.0, A79.8, A79.9, A80.9, A81.0, A81.8, A81.9, A82.0, A82.1, A82.9, A83.0, A83.6, A83.8, A83.9, A84.0, A84.8, A84.9, A85.0, A85.1, A85.2, A85.8, A87.0, A87.1, A87.2, A87.8, A87.9, A88.0, A88.8, A92.0, A92.3, A92.8, A92.9, A93.0, A93.8, A95.0, A95.1, A95.9, A96.0, A96.8, A96.9, A98.0, A98.3, A98.4, A98.8, B00.0, B00.1, B00.2, B00.3, B00.4, B00.5, B00.7, B00.8, B00.9, B01.0, B01.1, B01.2, B01.8, B01.9, B02.0, B02.1, B02.2, B02.3, B02.7, B02.8, B02.9, B05.0, B05.1, B05.2, B05.3, B05.4, B05.8, B05.9, B06.0, B06.8, B06.9, B08.0, B08.4, B08.5, B08.8, B16.0, B16.1, B16.2, B16.9, B17.0, B17.1, B17.8, B17.9, B18.0, B18.00, B18.09, B18.1, B18.10, B18.19, B18.2, B18.8, B18.9, B19.0, B19.9, B25.0, B25.1, B25.2, B25.8, B25.9, B26.0, B26.1, B26.2, B26.3, B26.8, B26.9, B27.0, B27.1, B27.8, B27.9, B30.0, B30.1, B30.2, B30.3, B30.8, B30.9, B33.0, B33.2, B33.3, B33.4, B33.8, B34.0, B34.1, B34.2, B34.3, B34.4, B34.8, B34.9, B35.9, B36.8, B37.0, B37.1, B37.2, B37.3, B37.4, B37.5, B37.6, B37.7, B37.8, B37.9, B38.0, B38.1, B38.2, B38.3, B38.4, B38.7, B38.8, B38.9, B41.0, B41.7, B41.8, B41.9, B43.1, B43.2, B44.0, B44.1, B44.2, B44.7, B44.8, B44.9, B45.0, B45.1, B45.2, B45.3, B45.7, B45.8, B45.9, B46.8, B47.1, B48.7, B48.8, B50.0, B50.8, B50.9, B51.0, B51.8, B51.9, B52.0, B52.8, B52.9, B53.0, B53.1, B53.8, B55.0, B55.1, B55.2, B55.9, B56.0, B56.1, B56.9, B58.0, B58.1, B58.2, B58.3, B58.8, B58.9, B60.0, B60.8, B65.0, B65.1, B65.2, B65.8, B65.9, B67.8, B67.9, B71.0, B71.8, B71.9, B76.1, B81.0, B81.4, B81.8, B82.0, B82.9, B83.0, B83.8, B83.9, B85.0, B85.1, B85.2, B85.4, B87.0, B87.1, B87.2, B87.3, B87.4, B87.8, B87.9, B88.0,

B94.0, B94.1, B94.2, B94.8, B94.9, B95.0, B95.1, B95.2, B95.3, B95.4, B95.5, B95.6, B95.7, B95.8, B96.0, B96.1, B96.2, B96.3, B96.4, B96.5, B96.7, B96.8, B97.0, B97.1, B97.2, B97.3, B97.4, B97.5, B97.6, B97.7, B97.8, D47.5, D73.3, E32.1, G00.0, G00.1, G00.2, G00.3, G00.8, G00.9, G02.0, G02.1, G02.8, G03.0, G03.1, G03.2, G03.8, G03.9, G04.2, G05.0, G05.1, G05.2, G06.0, G06.1, G06.2, G53.0, G53.1, G63.0, G73.1, G73.4, G93.3, G94.0, H03.0, H06.1, H10.0, H10.2, H10.3, H10.4, H10.5, H10.8, H10.9, H13.0, H13.1, H13.2, H16.1, H16.2, H19.1, H19.2, H19.3, H22.0, H32.0, H60.0, H60.1, H60.2, H60.3, H60.5, H60.8, H60.9, H62.0, H62.1, H62.3, H62.4, H65.0, H65.1, H65.2, H65.3, H65.4, H65.9, H66.0, H66.1, H66.2, H66.3, H66.4, H66.9, H67.0, H67.1, H67.8, H70.0, H70.1, H70.8, H70.9, H73.0, H73.1, H75.0, H94.0, I32.0, I32.1, I41.0, I41.1, I41.2, I42.3, I43.0, I52.0, I52.1, I68.1, I98.1, J01.0, J01.1, J01.2, J01.3, J01.4, J01.8, J01.9, J02.0, J02.8, J02.9, J03.0, J03.8, J03.9, J06.0, J06.8, J06.9, J10.0, J10.1, J10.8, J11.0, J11.1, J11.8, J12.0, J12.1, J12.2, J12.3, J12.8, J12.9, J15.0, J15.1, J15.2, J15.3, J15.4, J15.5, J15.6, J15.7, J15.8, J15.9, J16.0, J16.8, J17.0, J17.1, J17.2, J17.3, J17.8, J18.0, J18.1, J18.2, J18.8, J18.9, J20.0, J20.1, J20.2, J20.3, J20.4, J20.5, J20.6, J20.7, J21.0, J21.1, J31.0, J31.1, J31.2, J32.0, J32.1, J32.2, J32.3, J32.4, J32.8, J32.9, J34.0, J35.0, J39.0, J39.1, J44.0, J85.0, J85.1, J85.2, J85.3, K04.6, K04.7, K11.3, K12.2, K23.0, K35.2, K35.3, K35.8, K51.5, K52.3, K57.0, K57.1, K57.2, K57.3, K57.4, K57.5, K57.8, K57.9, K61.0, K61.1, K61.2, K61.3, K61.4, K63.0, K65.0, K65.8, K65.9, K67.0, K67.1, K67.2, K67.3, K75.0, K77.0, K80.0, K80.1, K80.2, K80.3, K80.36, K80.4, K80.5, K81.0, K81.1, K81.9, K83.0, K93.0, L02.0, L02.1, L02.2, L02.3, L02.4, L02.8, L02.9, L03.0, L03.1, L03.2, L03.3, L03.8, L03.9, L05.0, L05.9, L92.2, L98.3, M00.0, M00.00, M00.01, M00.02, M00.03, M00.04, M00.05, M00.06, M00.07, M00.08, M00.09, M00.1, M00.10, M00.11, M00.12, M00.13, M00.14, M00.15, M00.16, M00.17, M00.18, M00.19, M00.2, M00.20, M00.21, M00.22, M00.23, M00.24, M00.25, M00.26, M00.27, M00.28, M00.29, M00.8, M00.80, M00.81, M00.82, M00.83, M00.84, M00.85, M00.86, M00.87, M00.88, M00.89, M01.-, M01.0, M01.00, M01.01, M01.02, M01.03, M01.04, M01.05, M01.06, M01.07, M01.08, M01.09, M01.1, M01.10, M01.11, M01.12, M01.13, M01.14, M01.15, M01.16, M01.17, M01.18, M01.19, M01.2, M01.20, M01.21, M01.22, M01.23, M01.24, M01.25, M01.26, M01.27, M01.28, M01.29, M01.3, M01.30, M01.31, M01.32, M01.33, M01.34, M01.35, M01.36, M01.37, M01.38, M01.39, M01.4, M01.40, M01.41, M01.42, M01.43, M01.44, M01.45, M01.46, M01.47, M01.48, M01.49, M01.5, M01.50, M01.51, M01.52, M01.53, M01.54, M01.55, M01.56, M01.57, M01.58, M01.59, M01.6, M01.60, M01.61, M01.62, M01.63, M01.64, M01.65, M01.66, M01.67, M01.68, M01.69, M01.8, M01.80, M01.81, M01.82, M01.83, M01.84, M01.85, M01.86, M01.87, M01.88, M01.89, M02.3, M02.30, M02.31, M02.32, M02.33, M02.34, M02.35, M02.36, M02.37, M02.38, M02.39, M03.0, M03.00, M03.01, M03.02, M03.03, M03.04, M03.05, M03.06, M03.07, M03.08, M03.09, M03.1, M03.10, M03.11, M03.12, M03.13, M03.14, M03.15, M03.16, M03.17, M03.18, M03.19, M35.4, M46.2, M46.20, M46.21, M46.22, M46.23, M46.24, M46.25, M46.26, M46.27, M46.28, M46.29, M49.0, M49.00, M49.01, M49.02, M49.03, M49.04, M49.05, M49.06, M49.07, M49.08, M49.09, M49.1, M49.10, M49.11, M49.12, M49.13, M49.14, M49.15, M49.16, M49.17, M49.18, M49.19, M49.2, M49.20, M49.21, M49.22, M49.23, M49.24, M49.25, M49.26, M49.27, M49.28, M49.29, M49.3, M49.30, M49.31, M49.32, M49.33, M49.34, M49.35, M49.36, M49.37, M49.38, M49.39, M63.0, M63.1, M65.0, M65.00, M65.01, M65.02, M65.03, M65.04, M65.05, M65.06, M65.07, M65.08, M65.09, M68.0, M71.0, M71.00, M71.01, M71.02, M71.03, M71.04, M71.05, M71.06, M71.07, M71.08, M71.09, M73.0, M73.00, M73.01, M73.02, M73.03, M73.04, M73.05, M73.06, M73.07, M73.08, M73.09, M86.0, M86.00, M86.01, M86.02, M86.03, M86.04, M86.05, M86.06, M86.07, M86.08, M86.09, M86.1, M86.10, M86.11, M86.12, M86.13, M86.14, M86.15, M86.16, M86.17, M86.18, M86.19, M86.2, M86.20, M86.21, M86.22, M86.23, M86.24, M86.25, M86.26, M86.27, M86.28, M86.29, M86.3, M86.30, M86.31, M86.32, M86.33, M86.34, M86.35, M86.36, M86.37, M86.38, M86.39, M86.4, M86.40, M86.41, M86.42, M86.43, M86.44, M86.45, M86.46, M86.47, M86.48, M86.49, M86.5, M86.50, M86.51, M86.52, M86.53, M86.54, M86.55, M86.56, M86.57, M86.58, M86.59, M86.6, M86.60, M86.61, M86.62, M86.63, M86.64, M86.65, M86.66, M86.67, M86.68, M86.69, M86.8, M86.80, M86.81, M86.82, M86.83, M86.84, M86.85, M86.86, M86.87, M86.88, M86.89, M86.9, M86.90, M86.91, M86.92, M86.93, M86.94, M86.95, M86.96, M86.97, M86.98, M86.99, M89.6, M89.60, M89.61, M89.62, M89.63, M89.64, M89.65, M89.66, M89.67, M89.68, M89.69, N08.0, N15.1, N16.0, N22.0, N29.1, N33.0, N34.0, N35.1, N39.0, N41.2, N45.0, N45.9, N73.0, N73.1, N73.2, N73.3, N73.4, N73.5, N74.0, N74.1, N75.1, N76.4, N77.0, N77.1, O26.4, O35.3, O75.3, O98.0, O98.4, O98.5, O98.6, O98.8, O98.9, R57.2

Table S1. Definitions of ICD-10 code based variables and outcomes

Adapted from Drozd M. et al. (The Lancet Infectious Diseases, 2021 Aug;21(8):1184-1191. doi: 10.1016/S1473-3099(20)30978-6. Epub 2021 Mar 1.) and Kristensen S. et al. (The Journal of Rheumatology, 2024 Apr 1;51(4):350-359. doi: 10.3899/jrheum.2023-0907.)

Abbreviations: ASCVD: Atherosclerotic cardiovascular disease

Characteristics	All participants (n = 448 653)	hsCRP <1 mg/L (n = 177 007)	hsCRP 1–3 mg/L (n = 170 811)	hsCRP >3 mg/L (n = 100 835)
Asian or Asian British	8425 (1.9 %)	2794 (1.6 %)	3347 (2.0 %)	2284 (2.3 %)
Black or Black British	7102 (1.6 %)	2791 (1.6 %)	2484 (1.5 %)	1827 (1.8 %)
Chinese	1411 (0.3 %)	943 (0.5 %)	347 (0.2 %)	121 (0.1 %)
Do not know	195 (0.0 %)	69 (0.0 %)	82 (0.0 %)	44 (0.0 %)
Mixed	2675 (0.6 %)	1070 (0.6 %)	1008 (0.6 %)	597 (0.6 %)
Other ethnic group	4024 (0.9 %)	1588 (0.9 %)	1484 (0.9 %)	952 (0.9 %)
Unknown	1892 (0.4 %)	692 (0.4 %)	730 (0.4 %)	470 (0.5 %)
White	422 929 (94.3 %)	167 060 (94.4 %)	161 329 (94.4 %)	94 540 (93.8 %)

Table S2. Baseline ethnicity across hsCRP categories

Self-reported ethnicity in alphabetical order at baseline in the UK Biobank shown overall and stratified into hsCRP levels of <1 mg/L, 1–3 mg/L and >3 mg/L. Data are presented as numbers (percentage).

Ethnicity follows UK Biobank data-field 21000: Asian or Asian British (Indian, Pakistani, Bangladeshi, other Asian), Black or Black British (African, Caribbean, other Black), Chinese recorded separately, Mixed (White and Black Caribbean, White and Black African, White and Asian, Other mixed), Other ethnic group (any other specified), Unknown (missing/prefer not to answer), and Do not know as reported. White includes White British, White Irish, and other White background.

Abbreviations: hsCRP: high-sensitivity C-reactive protein

Characteristics	Missing (absolute, no.)
**Demographics**	
Age – years	0
Female sex – no. (%)	0
Ethnicity – no. (%)	463
**CV risk factors**	
BMI – kg/m^2	1661
Systolic blood pressure – mmHg	13 868
Hypertension – no. (%)	0
Dyslipidemia – no. (%)	0
Smoker – no. (%)	814
Type 2 diabetes mellitus – no. (%)	0
SMuRF-less – no. (%)	14 679
**Laboratory**	
Total cholesterol – mg/dL	142
HDL cholesterol – mg/dL	37 981
LDL cholesterol – mg/dL	915
Creatinine – mg/dL	344
eGFR – ml/min/1,73m^2	344
HbA1c – %	23 106
hsCRP – mg/L	0

Table S3. Missing data for baseline variables

Absolute numbers of missing baseline demographic, clinical and laboratory characteristics.

Abbreviations: BMI: body mass index; CV: cardiovascular; eGFR: estimated glomerular filtration rate; HbA1c: glycated hemoglobin A1c; HDL cholesterol: high-density lipoprotein cholesterol; hsCRP: high-sensitivity C-reactive protein; LDL cholesterol: low-density lipoprotein cholesterol; SMuRF-less: without Standard Modifiable cardiovascular Risk Factors

Endpoint	hsCRP group (mg/L)	*n*	No of events	Crude HR# (95% CI)	Adj. HR*# (95% CI)	AR** (%) (95% CI)
MACE	<1	170 755	6641	Ref.	Ref.	2.20 (2.12, 2.29)
	1–3	164 461	9268	1.31 (1.30, 1.33)	1.16 (1.14, 1.18)	2.55 (2.46, 2.65)
	>3	96 205	6677	1.73 (1.68, 1.77)	1.35 (1.31, 1.39)	2.96 (2.85, 3.08)
	<2	280 801	12 537	Ref.	Ref.	2.32 (2.24, 2.41)
	≥2	150 620	10 049	1.45 (1.42, 1.47)	1.22 (1.20, 1.25)	2.84 (2.73, 2.95)
CV death	<1	170 755	1480	Ref.	Ref.	0.28 (0.26, 0.31)
	1–3	164 461	2280	1.50 (1.46, 1.54)	1.27 (1.23, 1.30)	0.36 (0.33, 0.39)
	>3	96 205	2100	2.25 (2.14, 2.37)	1.61 (1.52, 1.71)	0.45 (0.42, 0.49)
	<2	280 801	2894	Ref.	Ref.	0.31 (0.28, 0.33)
	≥2	150 620	2966	1.73 (1.67, 1.79)	1.38 (1.32, 1.43)	0.42 (0.39, 0.46)
All-cause death	<1	170 755	9668	Ref.	Ref.	2.60 (2.52, 2.68)
	1–3	164 461	13 096	1.40 (1.39, 1.42)	1.27 (1.26, 1.29)	3.29 (3.20, 3.38)
	>3	96 205	11 397	1.98 (1.94, 2.02)	1.62 (1.58, 1.66)	4.17 (4.04, 4.30)
	<2	280 801	17 842	Ref.	Ref.	2.83 (2.75, 2.92)
	≥2	150 620	16 319	1.58 (1.56, 1.61)	1.38 (1.36, 1.40)	3.89 (3.78, 4.01)

Table S4. Relative and absolute risk estimates for the association of hsCRP with CV outcomes in 431 421 individuals (Model 1)

Uni- and multivariable Cox proportional hazards models assessing the association of hsCRP with CV outcomes (MACE, CV death and all-cause death) during the observation period in individuals without known ASCVD. Hazard ratios (HR) and absolute risks (AR) with 95% confidence intervals (CI) are provided for hsCRP levels categorized into groups of <1 mg/L, 1–3 mg/L, and >3 mg/L as well as dichotomized at 2 mg/L derived from established cut-offs from guidelines and previous studies. Uni- and multivariable models were performed within the multivariable model cohort. The <1 mg/L and <2 mg/L groups serve as reference for respective comparisons.

# All p-values were <0.001.

* Multivariable model adjusted for age, sex, body mass index, type 2 diabetes mellitus, smoking, systolic blood pressure, LDL-cholesterol, creatinine and hsCRP.

** Absolute risks were computed using the multivariable Cox regression models, with the risks assessed over the entire follow-up period.

Abbreviations: AR: absolute risk; CV: cardiovascular; hsCRP: high-sensitivity C-reactive protein; MACE: major adverse cardiovascular events; Ref.: Reference value

Model	Endpoint	*n*	No of events	Interquartile HR hsCRP (95% CI)	p-value*
Model 3	MACE	431 421	22 586	1.21 (1.19, 1.24)	<0.001
	CV death		5860	1.35 (1.30, 1.40)	
	All-cause death		35,299	1.36 (1.34, 1.38)	
Model 4	MACE	349 725	17 922	1.20 (1.18, 1.23)	
	CV death		4538	1.35 (1.29, 1.41)	
	All-cause death		26 579	1.31 (1.29, 1.33)	

Table S5. Cox regression analyses assessing hsCRP and the risk of CV outcomes (Model 3 and Model 4)

Multivariable Cox proportional hazards models with interquartile hazard ratios (HR) and 95% confidence intervals (CI) assessing the association of hsCRP with CV outcomes (MACE, CV death and all-cause death) in individuals without known ASCVD.

Model 3: Model 1, with eGFR instead of serum creatinine.
Model 4: Model 2, with eGFR instead of serum creatinine.
* P-values refer to the statistical significance of the reported hazard ratios for hsCRP levels in relation to each endpoint.

Abbreviations: ASCVD: atherosclerotic cardiovascular disease; CV: cardiovascular; eGFR: estimated glomerular filtration rate; hsCRP: high-sensitivity C-reactive protein; MACE: major adverse cardiovascular events

Endpoint	hsCRP group (mg/L)	*n*	No of events	Adj. HR*# (95% CI)	AR** (%) (95% CI)
MACE	<1	170 755	6641	Ref.	2.13 (2.05, 2.21)
	1–3	164 461	9268	1.16 (1.14, 1.18)	2.46 (2.38, 2.55)
	>3	96 205	6677	1.35 (1.31, 1.39)	2.85 (2.74, 2.96)
	<2	280 801	12 537	Ref.	2.24 (2.16, 2.33)
	≥2	150 620	10 049	1.22 (1.20, 1.25)	2.73 (2.63, 2.84)
CV death	<1	170 755	1480	Ref.	0.27 (0.25, 0.29)
	1–3	164 461	2280	1.26 (1.23, 1.30)	0.34 (0.32, 0.37)
	>3	96 205	2100	1.59 (1.50, 1.69)	0.43 (0.40, 0.46)
	<2	280 801	2894	Ref.	0.29 (0.27, 0.32)
	≥2	150 620	2966	1.37 (1.32, 1.42)	0.40 (0.37, 0.43)
All-cause death	<1	170 755	9668	Ref.	2.58 (2.51, 2.66)
	1–3	164 461	13 096	1.27 (1.25, 1.28)	3.26 (3.17, 3.35)
	>3	96 205	11 397	1.60 (1.57, 1.64)	4.11 (3.99, 4.24)
	<2	280 801	17842	Ref.	2.81 (2.73, 2.89)
	≥2	150 620	16319	1.38 (1.35, 1.40)	3.84 (3.73, 3.95)

Table S6. Multivariable relative and absolute risk estimates for the association of hsCRP with CV outcomes (Model 3)

Multivariable Cox proportional hazards models assessing the association of hsCRP with CV outcomes (MACE, CV death and all-cause death) during the observation period in individuals without known ASCVD. Hazard ratios (HR) and absolute risks (AR) with 95% confidence intervals (CI) are provided for hsCRP levels categorized into groups of <1 mg/L, 1–3 mg/L, and >3 mg/L as well as dichotomized at 2 mg/L derived from established cut-offs from guidelines and previous studies. Multivariable models were performed within the multivariable model cohort. The <1 mg/L and <2 mg/L groups serve as reference for respective comparisons.

[#] All p-values were <0.001.

* Multivariable model adjusted for age, sex, body mass index, type 2 diabetes mellitus, smoking, systolic blood pressure, LDL-C, eGFR and hsCRP.

** Absolute risks were computed using the multivariable Cox regression models, with the risks assessed over the entire follow-up period.

Abbreviations: AR: absolute risk; ASCVD: Atherosclerotic cardiovascular disease; CV: cardiovascular; eGFR: estimated glomerular filtration rate; hsCRP: high-sensitivity C-reactive protein; LDL-C: low-density lipoprotein cholesterol; MACE: major adverse cardiovascular events; Ref.: Reference value

Endpoint	hsCRP group (mg/L)	*n*	No of events	Adj. HR*# (95% CI)	AR** (%) (95% CI)
MACE	<1	142 554	5458	Ref.	1.91 (1.83, 2.00)
	1–3	132 859	7335	1.16 (1.14, 1.18)	2.21 (2.12, 2.30)
	>3	74 312	5129	1.34 (1.29, 1.38)	2.55 (2.43, 2.66)
	<2	232 143	10 171	Ref.	2.02 (1.93, 2.10)
	≥2	117 582	7751	1.21 (1.19, 1.24)	2.44 (2.34, 2.55)
CV death	<1	142 554	1172	Ref.	0.24 (0.22, 0.26)
	1–3	132 859	1789	1.26 (1.22, 1.31)	0.30 (0.27, 0.33)
	>3	74 312	1577	1.60 (1.49, 1.71)	0.38 (0.34, 0.41)
	<2	232 143	2288	Ref.	0.26 (0.24, 0.28)
	≥2	117 582	2250	1.37 (1.31, 1.43)	0.35 (0.32, 0.38)
All-cause death	<1	142 554	7766	Ref.	2.34 (2.26, 2.42)
	1–3	132 859	10 259	1.24 (1.22, 1.25)	2.89 (2.80, 2.98)
	>3	74 312	8554	1.53 (1.49, 1.57)	3.56 (3.44, 3.68)
	<2	232 143	14 222	Ref.	2.53 (2.44, 2.61)
	≥2	117 582	12 357	1.33 (1.30, 1.35)	3.34 (3.23, 3.45)

Table S7. Multivariable relative and absolute risk estimates for the association of hsCRP with CV outcomes (Model 4)

Multivariable Cox proportional hazards models assessing the association of hsCRP with CV outcomes (MACE, CV death and all-cause death) during the observation period in individuals without known ASCVD. Hazard ratios (HR) and absolute risks (AR) with 95% confidence intervals (CI) are provided for hsCRP levels categorized into groups of <1 mg/L, 1–3 mg/L, and >3 mg/L as well as dichotomized at 2 mg/L derived from established cut-offs from guidelines and previous studies. Multivariable models were performed within the multivariable model cohort. The <1 mg/L and <2 mg/L groups serve as reference for respective comparisons.

# All p-values were <0.001.

* Multivariable model adjusted for age, sex, body mass index, type 2 diabetes mellitus, smoking, systolic blood pressure, LDL-C, eGFR, hsCRP, infectious diseases, inflammatory bowel diseases, autoimmune diseases, malignant diseases, rheumatoid arthritis, psoriasis, atrial fibrillation and flutter, aortic stenosis, heart failure, MET (summed MET minutes per week for all activity), corticosteroids, immunosuppressants, statins, RAASi and CCB

** Absolute risks were computed using the multivariable Cox regression models, with the risks assessed over the entire follow-up period.

Abbreviations: AR: absolute risk; ASCVD: Atherosclerotic cardiovascular disease; CCB: calcium channel blockers; CV: cardiovascular; eGFR: estimated glomerular filtration rate; hsCRP: high-sensitivity C-reactive protein; LDL-C: low-density lipoprotein cholesterol; MACE: major adverse cardiovascular events; MET: Metabolic Equivalent Task, RAASi: renin-angiotensin-aldosterone system inhibitors; Ref.: Reference value

Characteristics	All participants (n = 448 653)	hsCRP <1 mg/L (n = 177 007)	hsCRP 1–3 mg/L (n = 170 811)	hsCRP >3 mg/L (n = 100 835)
Corticosteroids	3540 (0.8 %)	848 (0.5 %)	1214 (0.7 %)	1478 (1.5 %)
Immunosuppressants	3200 (0.7 %)	610 (0.3 %)	1050 (0.6 %)	1540 (1.5 %)
Statins	61 233 (13.6 %)	21 562 (12.2 %)	24 847 (14.5 %)	14 824 (14.7 %)
RAASi	53 867 (12.0 %)	15 474 (8.7 %)	21 796 (12.8 %)	16 597 (16.5 %)
CCB	9843 (2.2 %)	2646 (1.5 %)	4030 (2.4 %)	3167 (3.1 %)

Table S8. Baseline medication across hsCRP categories

Baseline medication use included in multivariable models (Model 2 and Model 4), shown overall and stratified by hsCRP levels (<1 mg/L, 1–3 mg/L and >3 mg/L). Data are presented as numbers (percentage).

Abbreviations: CCB: calcium channel blockers; hsCRP: high-sensitivity C-reactive protein; RAASi: renin-angiotensin-aldosterone system inhibitors

(A)

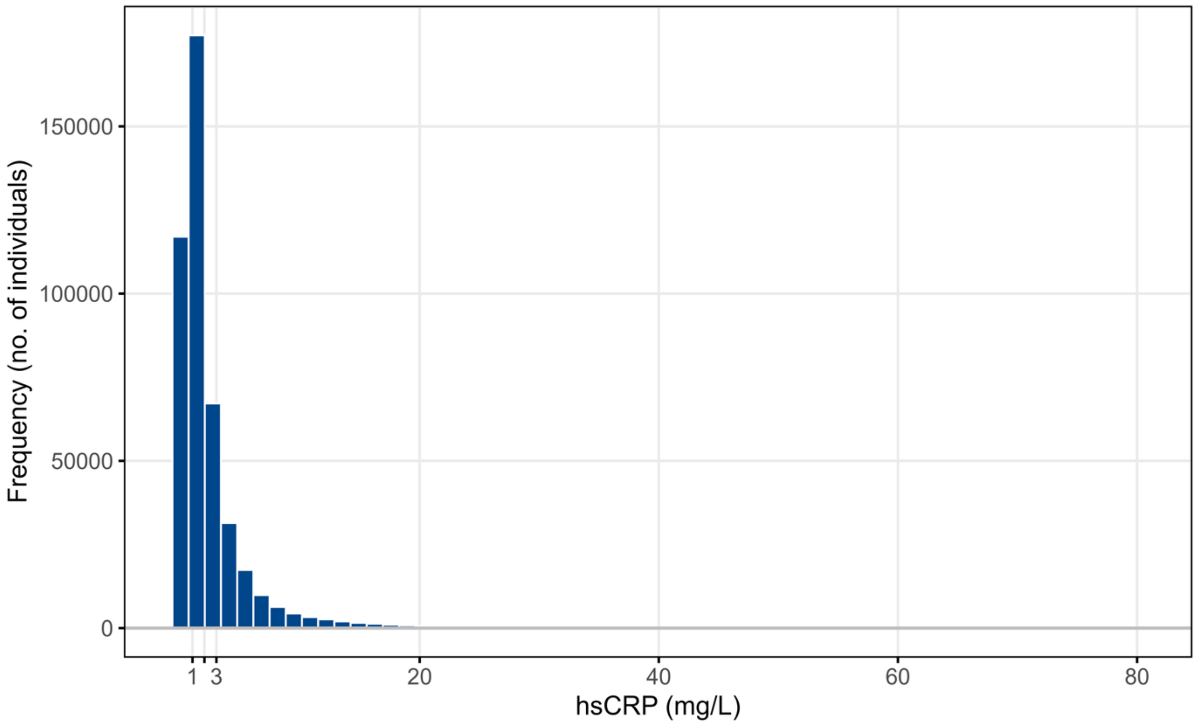

(B)

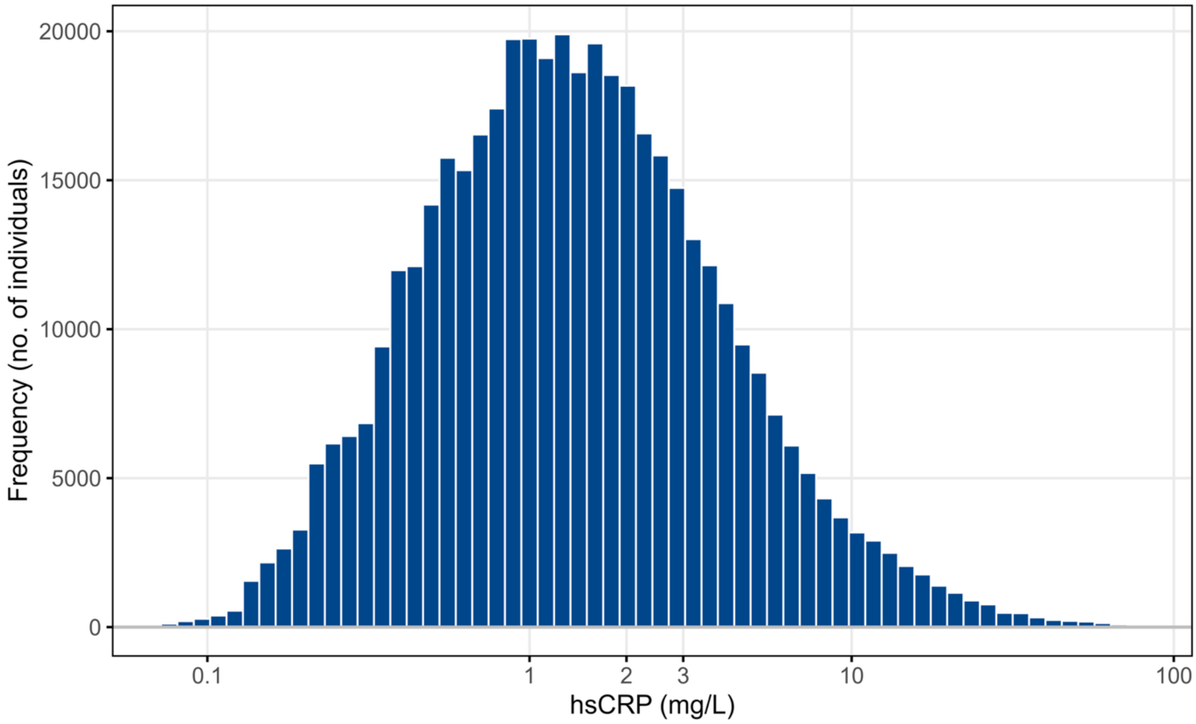

Figure S1. Distribution of hsCRP levels at baseline

Histograms visualizing the distribution of hsCRP levels among 448 653 participants without known ASCVD. Median hsCRP levels were 1.32 [0.65, 2.74] mg/L. hsCRP values are plotted against absolute frequencies (number of individuals). Empirical cut-off values of 1, 2 and 3 mg/L are highlighted.

(A) Distribution on a linear scale.

(B) Distribution on a logarithmically transformed x-axis.

Abbreviations: hsCRP: high-sensitivity C-reactive protein; ASCVD: Atherosclerotic cardiovascular disease

(A)

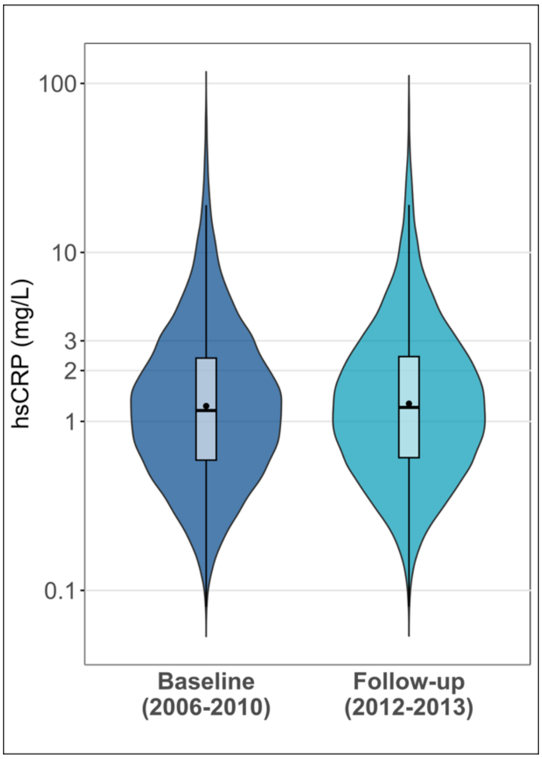

hsCRP (mg/L)
100
10
3
2
1
0.1
Baseline
(2006-2010)
Follow-up
(2012-2013)

(B)

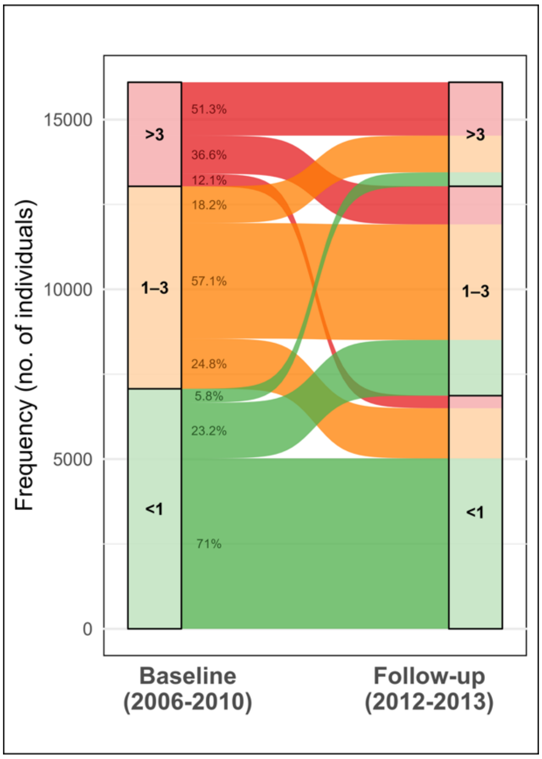

Frequency (no. of individuals)
15000
10000
5000
0
>3
1–3
<1
51.3%
36.6%
12.1%
18.2%
57.1%
24.8%
5.8%
23.2%
71%
Baseline
(2006-2010)
Follow-up
(2012-2013)

(C)

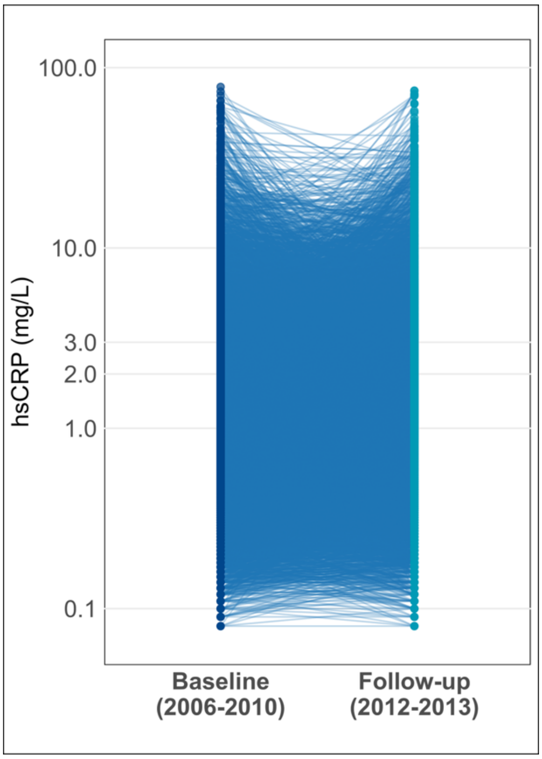

hsCRP (mg/L)
100.0
10.0
3.0
2.0
1.0
0.1
Baseline
(2006-2010)
Follow-up
(2012-2013)

(D)

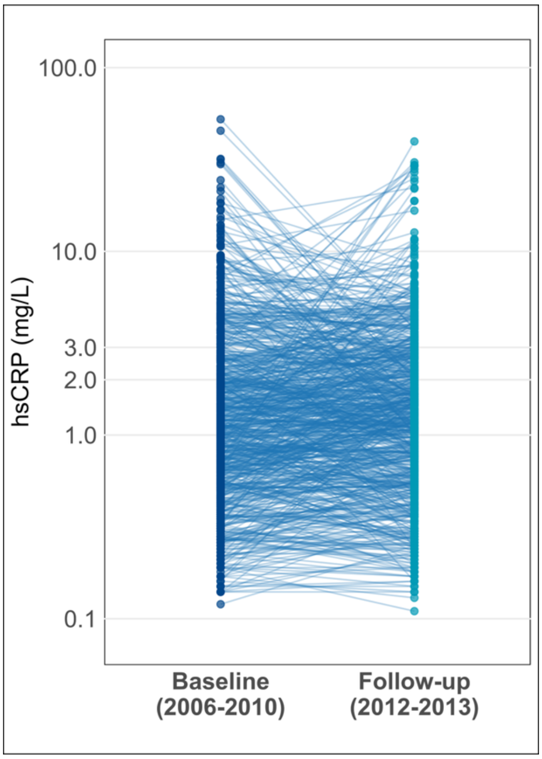

hsCRP (mg/L)
100.0
10.0
3.0
2.0
1.0
0.1
Baseline
(2006-2010)
Follow-up
(2012-2013)

(E)

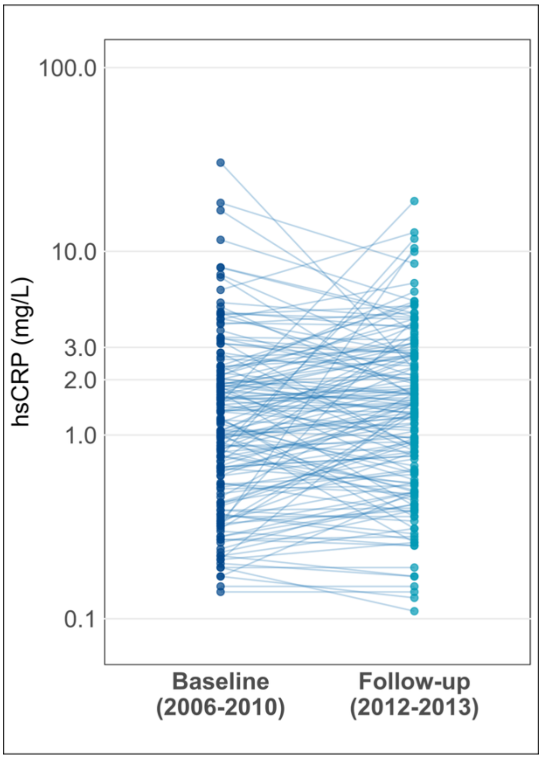

Figure S2. Long-term stability in serial hsCRP measurements

Graphical display of hsCRP levels at baseline (2006–2010) and follow-up (2012–2013) over a median follow-up period of 4.4 [3.7, 4.9] years within the subset of participants with available serial measurements (n=15 967).

(A) Violin plot showing the distribution of hsCRP levels during both time points, displayed on a logarithmic scale.

(B) Alluvial plot illustrating the distribution and transition between predefined hsCRP categories (<1 mg/L, 1–3 mg/L, >3 mg/L). Percentages indicate the proportion of participants within each baseline category who remained stable or shifted to another category at follow-up.

(C) Flowline (spaghetti) plot showing individual hsCRP trajectories from baseline to follow-up for all participants.

(D) Flowline (spaghetti) plot showing individual hsCRP trajectories from baseline to follow-up for a random 5% subset of the cohort.

(E) Flowline (spaghetti) plot showing individual hsCRP trajectories from baseline to follow-up for a random 1% subset of the cohort, to illustrate detailed intra-individual changes.

Abbreviations: hsCRP: high-sensitivity C-reactive protein

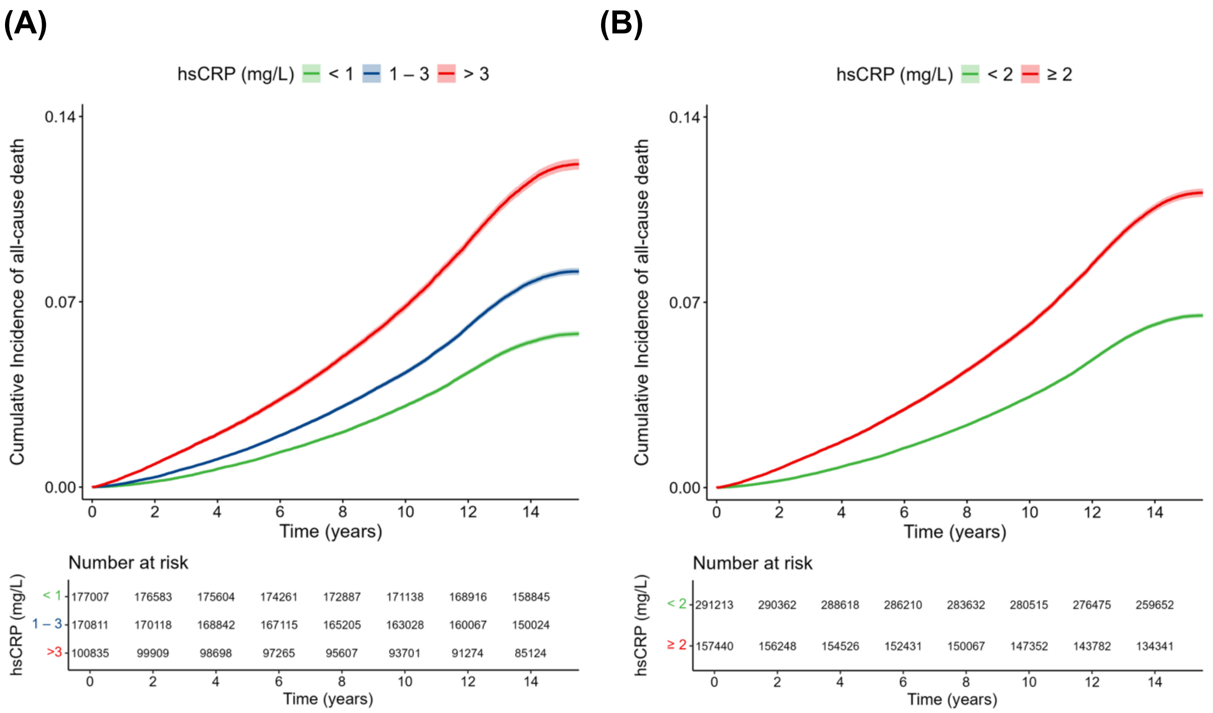

Figure S3. Association of hsCRP with all-cause death

Kaplan-Meier curves illustrating the cumulative incidence of all-cause death stratified by hsCRP levels with the number at risk displayed below the x-axis. Shaded areas represent 95% confidence intervals.

(A) Incidence of all-cause death comparing levels of hsCRP <1 mg/L vs. 1-3 mg/L vs. >3 mg/L.

(B) Incidence of all-cause death comparing levels of hsCRP <2 mg/L vs. ≥2 mg/L.

All log-rank P-values <0.001.

Abbreviation: hsCRP: high-sensitivity C-reactive protein

(A)

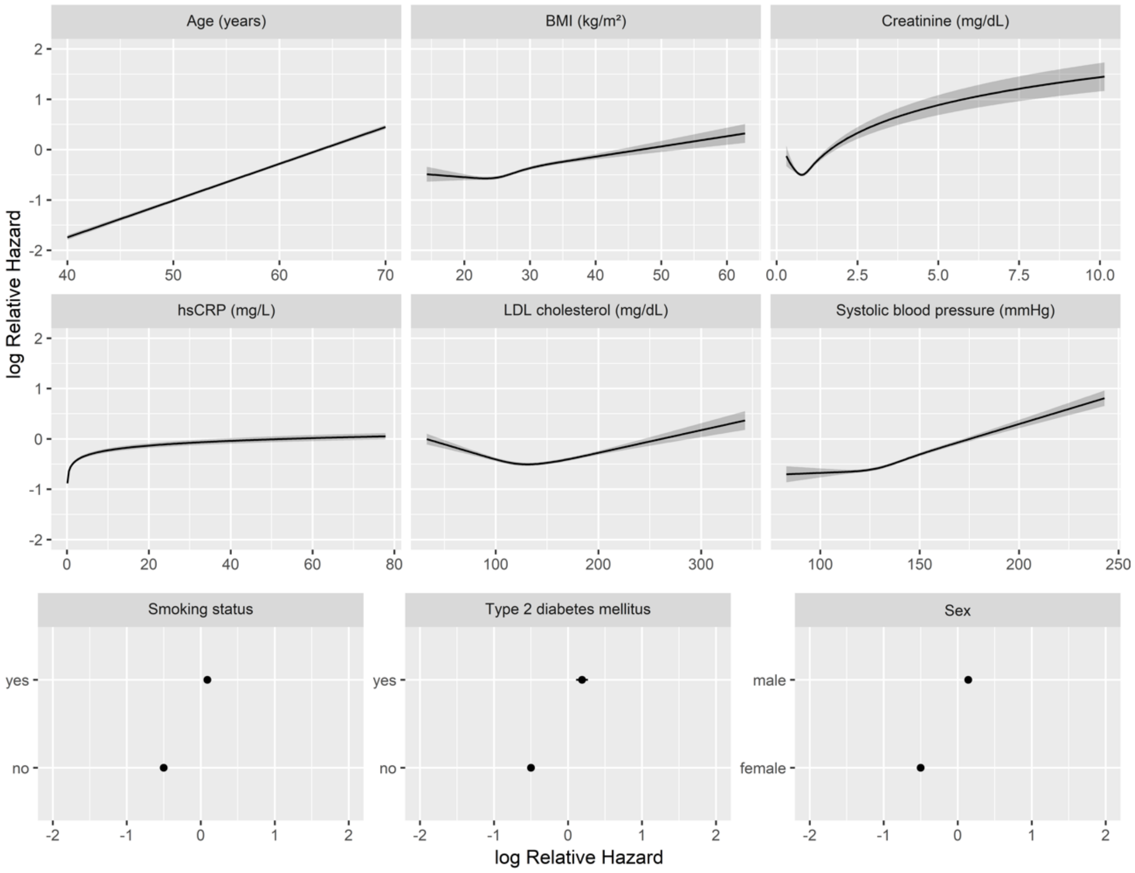
Age (years)
BMI (kg/m²)
Creatinine (mg/dL)
hsCRP (mg/L)
LDL cholesterol (mg/dL)
Systolic blood pressure (mmHg)
log Relative Hazard
Smoking status
Type 2 diabetes mellitus
Sex
yes
no
male
female
log Relative Hazard

(B)

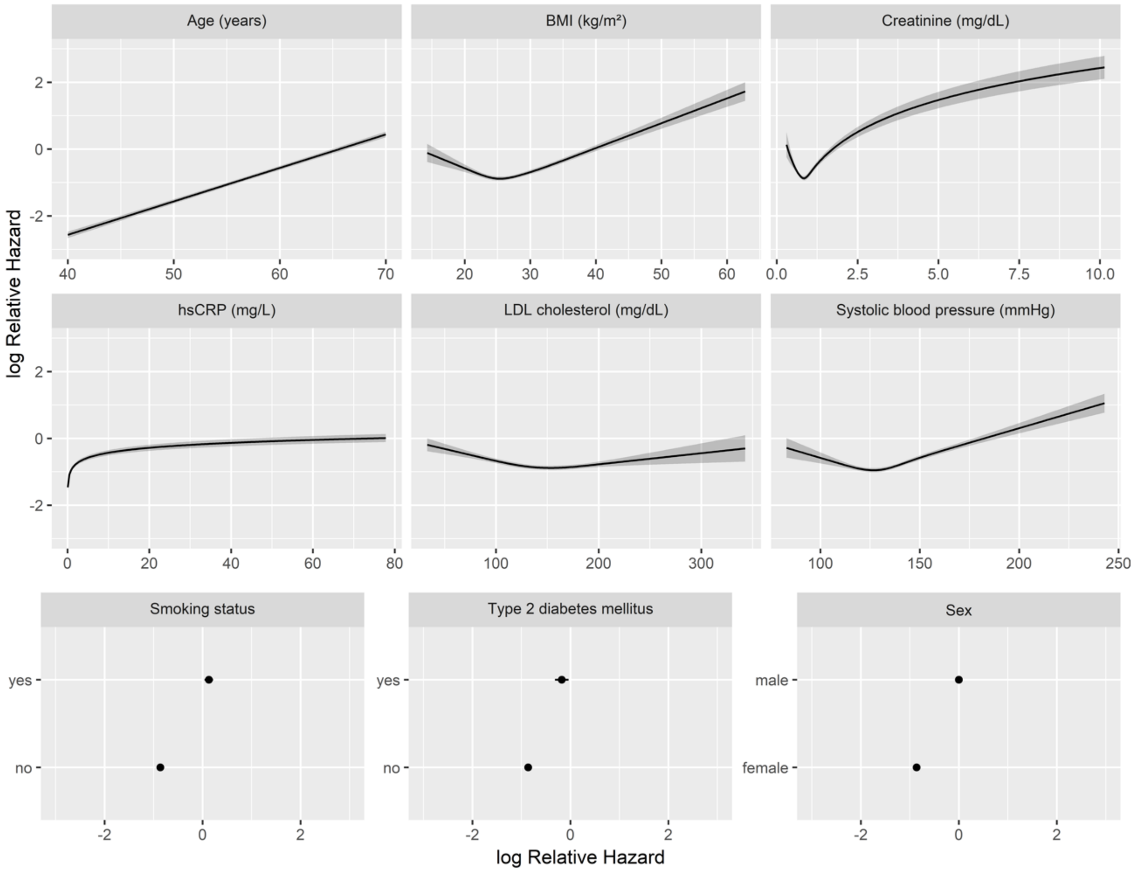
Age (years)
BMI (kg/m²)
Creatinine (mg/dL)
hsCRP (mg/L)
LDL cholesterol (mg/dL)
Systolic blood pressure (mmHg)
log Relative Hazard
Smoking status
Type 2 diabetes mellitus
Sex
yes
no
male
female
log Relative Hazard

(C)

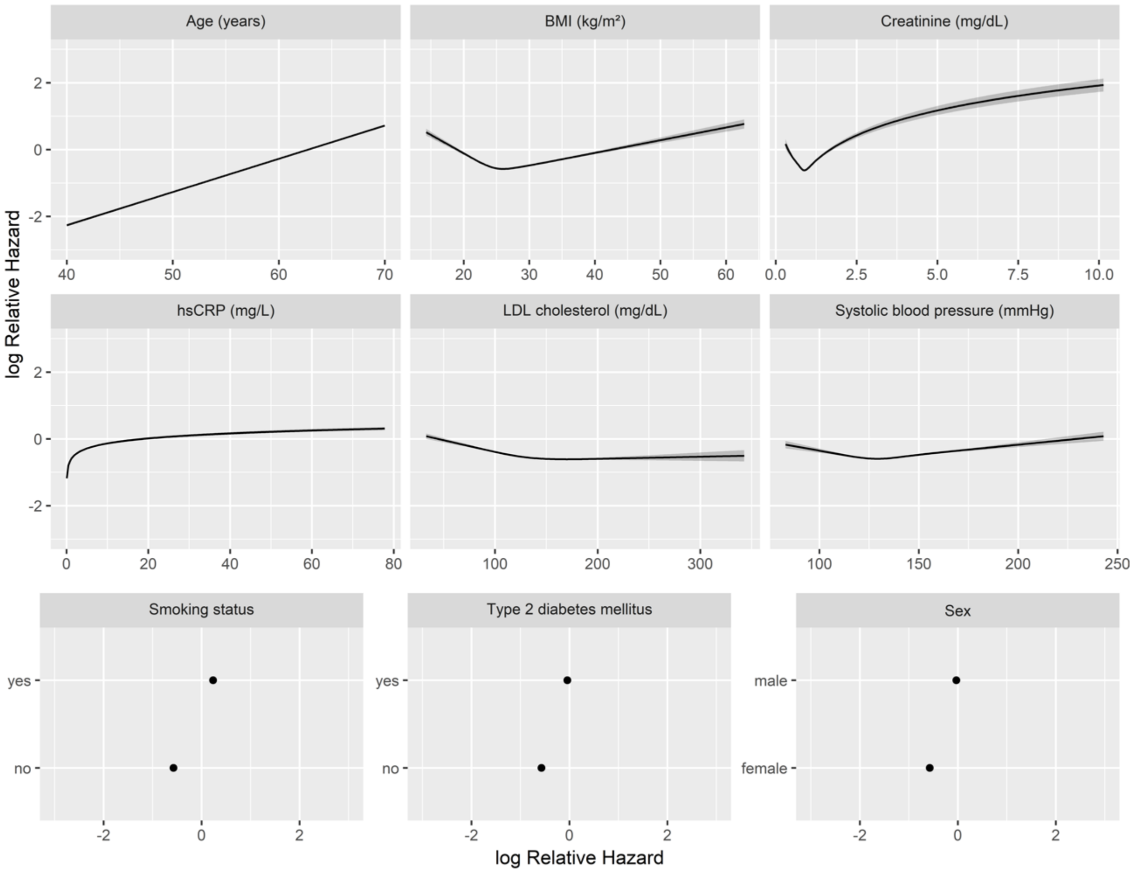

Figure S4. Partial effect plots for associations of clinical variables with CV outcomes (Model 1)

Partial effect plots with corresponding log relative hazard estimates illustrating the associations of age, BMI, hsCRP, creatinine, LDL-C, systolic blood pressure, smoking, diabetes and sex with risk in individuals without known ASCVD. BMI, systolic blood pressure, LDL-C and creatinine were modeled using restricted cubic splines, while creatinine and hsCRP were log-transformed and included as log-transformed continuous predictors as spline modeling did not improve model fit. Categorical variables (smoking, diabetes, and sex) are represented as points with corresponding log relative hazard estimates.

(A) Association with MACE.

(B) Association with CV death.

(C) Association with all-cause death.

Abbreviations: ASCVD: Atherosclerotic cardiovascular disease; BMI: body mass index; CV: Cardiovascular; hsCRP: high-sensitivity C-reactive protein; LDL-C: low-density lipoprotein cholesterol; MACE: Major adverse cardiovascular events

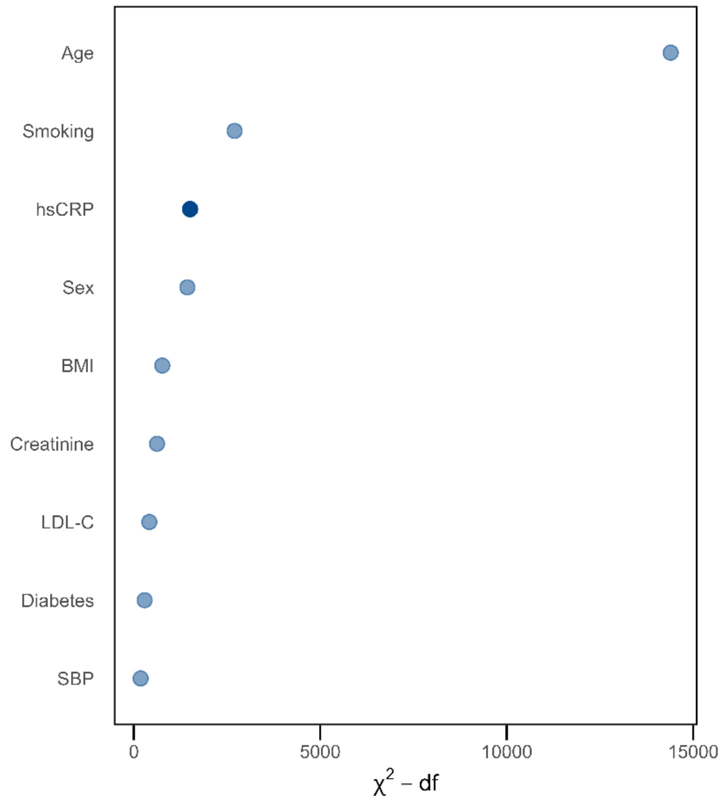

Figure S5. Variable importance of hsCRP in risk prediction of all-cause death (Model 1)

Variable importance plots from multivariable Cox regression models assessing the individual contribution of hsCRP and additional CV risk factors, including age (years), sex, BMI (kg/m^2), diabetes, smoking, systolic blood pressure (mmHg), LDL-C (mg/dL) and creatinine (mg/dL) to risk prediction of all-cause death. The likelihood ratio (LR) Chi² statistic minus the degrees of freedom (Chi² - df) is plotted for each model variable.

Abbreviations: hsCRP: high-sensitivity C-reactive protein; BMI: body mass index; LDL-C: low-density lipoprotein cholesterol; mmHg: millimeters of mercury; SBP: systolic blood pressure

(A)

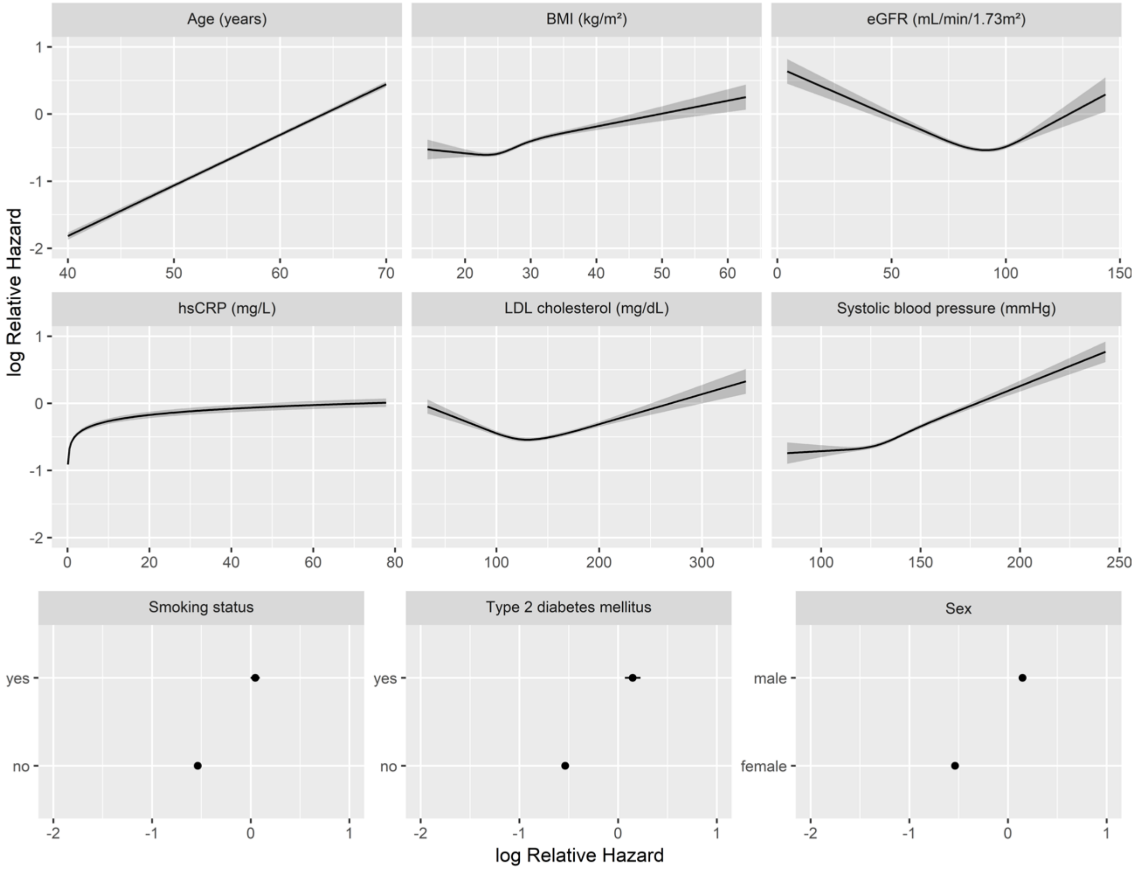
Age (years)
BMI (kg/m²)
eGFR (mL/min/1.73m²)
hsCRP (mg/L)
LDL cholesterol (mg/dL)
Systolic blood pressure (mmHg)
Smoking status
Type 2 diabetes mellitus
Sex
yes
no
male
female
log Relative Hazard
log Relative Hazard

(B)

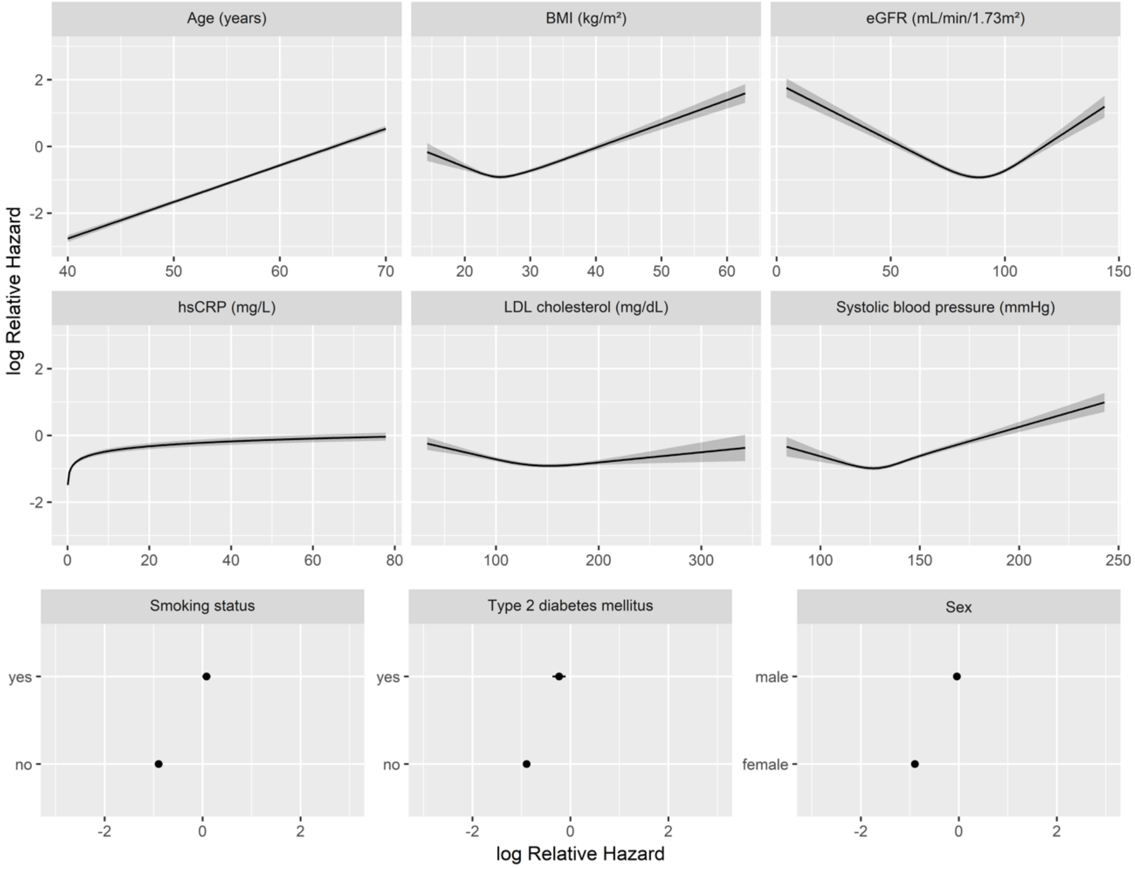
Age (years)
BMI (kg/m²)
eGFR (mL/min/1.73m²)
hsCRP (mg/L)
LDL cholesterol (mg/dL)
Systolic blood pressure (mmHg)
Smoking status
Type 2 diabetes mellitus
Sex
yes
no
male
female
log Relative Hazard
log Relative Hazard

(C)

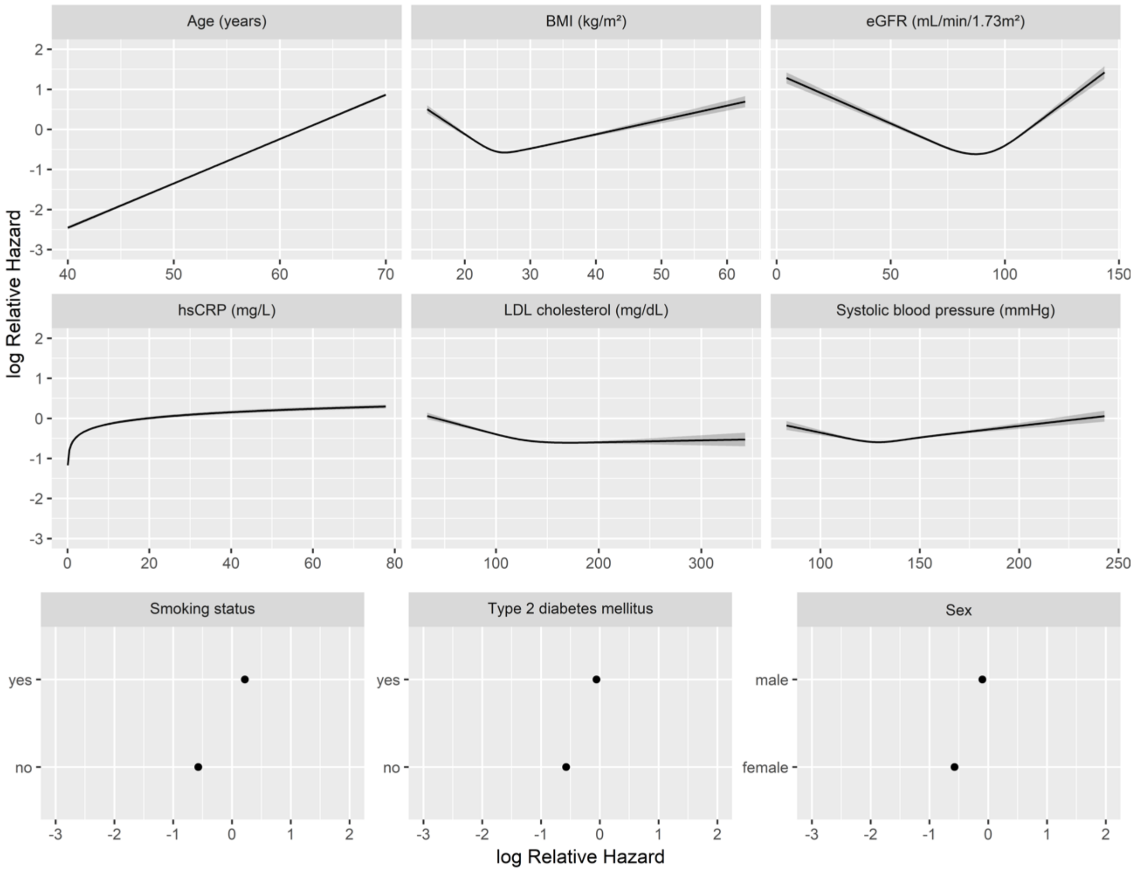

Figure S6. Partial effect plots for associations of clinical variables with CV outcomes (Model 3)

Partial effect plots with corresponding log relative hazard estimates illustrating the associations of age, BMI, hsCRP, creatinine, LDL-C, systolic blood pressure, smoking, diabetes and sex with risk in individuals without known ASCVD. BMI, systolic blood pressure, LDL-C and eGFR were modeled using restricted cubic splines, while creatinine and hsCRP were log-transformed and included as log-transformed continuous predictors as spline modeling did not improve model fit. Categorical variables (smoking, diabetes, and sex) are represented as points with corresponding log relative hazard estimates.

(A) Association with MACE.

(B) Association with CV death.

(C) Association with all-cause death.

Abbreviations: ASCVD: Atherosclerotic cardiovascular disease; BMI: body mass index; CV: Cardiovascular; eGFR: estimated glomerular filtration rate; hsCRP: high-sensitivity C-reactive protein; LDL-C: low-density lipoprotein cholesterol; MACE: Major adverse cardiovascular events

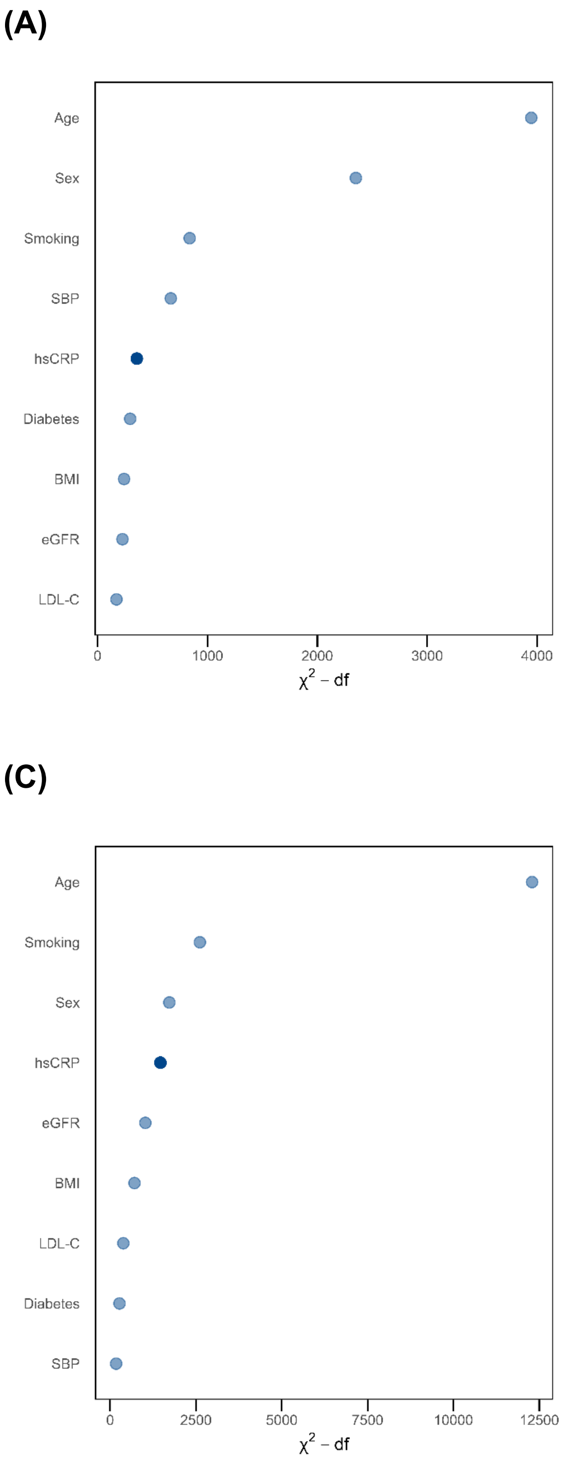

Figure S7. Variable importance of hsCRP in risk prediction of MACE, CV death and all-cause death (Model 3)

Variable importance plots from multivariable Cox regression models assessing the individual contribution of hsCRP and additional CV risk factors, including age (years), sex, BMI (kg/m2), diabetes, smoking, systolic blood pressure (mmHg), LDL-C (mg/dL) and eGFR (ml/min/1.73m^2) to risk prediction of all-cause death. The likelihood ratio (LR) Chi² statistic minus the degrees of freedom (Chi² - df) is plotted for each model variable.

(A) Variable importance for prediction of MACE.

(B) Variable importance for prediction of CV death.

(C) Variable importance for prediction of all-cause death.

Abbreviations: BMI: body mass index; CV: cardiovascular; eGFR: estimated glomerular filtration rate; hsCRP: high-sensitivity C-reactive protein; LDL-C: low-density lipoprotein cholesterol; MACE: major adverse cardiovascular events; mmHg: millimeters of mercury; SBP: systolic blood pressure

(A)

Subgroup	adj. HR (95%-CI)	p-value
**Age (years)**		
≤58 (median)	1.57 (1.46, 1.70)	<0.001
>58 (median)	1.28 (1.20, 1.37)	
**Sex**		
female	1.41 (1.32, 1.50)	0.670
male	1.43 (1.35, 1.51)	
**BMI (kg/m²)**		
<25	1.51 (1.41, 1.61)	<0.001
≥25 - 30	1.38 (1.30, 1.47)	
≥30	1.21 (1.12, 1.30)	
**Type 2 diabetes mellitus**		
No	1.41 (1.32, 1.50)	0.005
Yes	1.75 (1.48, 2.07)	
**Hypertension**		
No	1.41 (1.32, 1.50)	0.151
Yes	1.51 (1.35, 1.68)	
**Smoking status**		
No	1.41 (1.32, 1.50)	<0.001
Yes	1.74 (1.58, 1.91)	
**eGFR (mL/min/1.73m²)**		
<60	1.47 (1.33, 1.63)	0.285
≥60	1.41 (1.32, 1.50)	
**LDL cholesterol (mg/dL)**		
<100	1.25 (1.15, 1.36)	0.002
≥100 - 116	1.32 (1.22, 1.42)	
>116 - 150	1.40 (1.31, 1.49)	
>150	1.45 (1.35, 1.56)	
**Dyslipidemia**		
No	1.41 (1.32, 1.50)	0.954
Yes	1.40 (1.19, 1.66)	

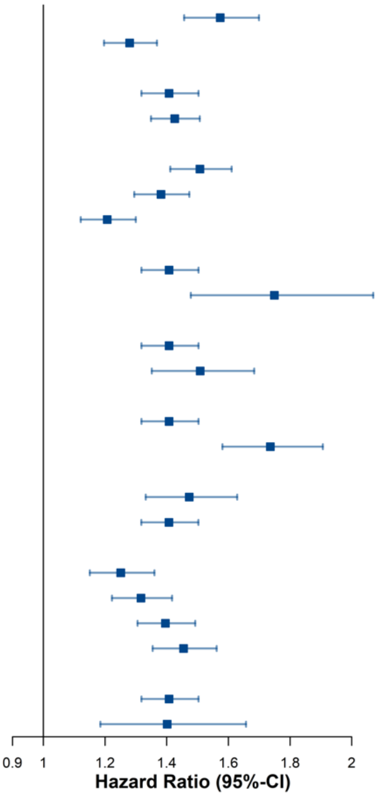

(B)

Subgroup	adj. HR (95%-CI)	p-value
**Age (years)**		
≤58 (median)	1.25 (1.21, 1.30)	<0.001
>58 (median)	1.13 (1.09, 1.17)	
**Sex**		
female	1.19 (1.15, 1.22)	0.670
male	1.19 (1.16, 1.23)	
**BMI (kg/m²)**		
<25	1.23 (1.19, 1.27)	<0.001
≥25 - 30	1.17 (1.14, 1.21)	
≥30	1.10 (1.06, 1.14)	
**Type 2 diabetes mellitus**		
No	1.19 (1.15, 1.22)	0.005
Yes	1.32 (1.21, 1.44)	
**Hypertension**		
No	1.19 (1.15, 1.22)	0.151
Yes	1.23 (1.16, 1.30)	
**Smoking status**		
No	1.19 (1.15, 1.22)	<0.001
Yes	1.32 (1.26, 1.38)	
**eGFR (mL/min/1.73m²)**		
<60	1.21 (1.15, 1.27)	0.285
≥60	1.19 (1.15, 1.22)	
**LDL cholesterol (mg/dL)**		
<100	1.12 (1.07, 1.17)	0.002
≥100 - 116	1.15 (1.11, 1.19)	
>116 - 150	1.18 (1.14, 1.22)	
>150	1.21 (1.16, 1.25)	
**Dyslipidemia**		
No	1.19 (1.15, 1.22)	0.954
Yes	1.18 (1.09, 1.29)	

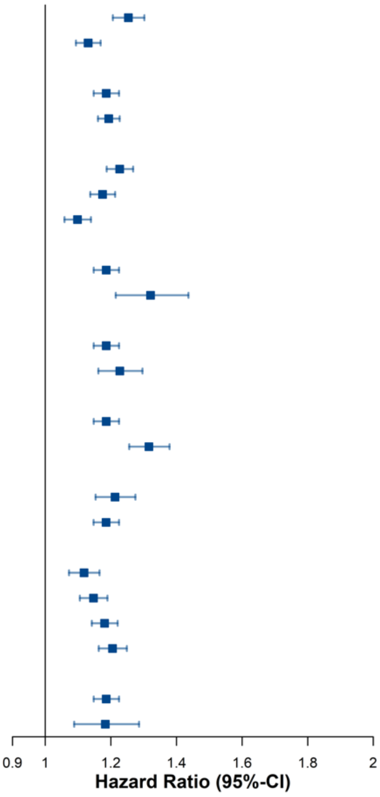

(C)

Subgroup	adj. HR (95%-CI)	p-value
**Age (years)**		
≤58 (median)	1.26 (1.21, 1.31)	<0.001
>58 (median)	1.13 (1.09, 1.17)	
**Sex**		
female	1.19 (1.15, 1.23)	0.670
male	1.20 (1.16, 1.23)	
**BMI (kg/m²)**		
<25	1.23 (1.19, 1.27)	<0.001
≥25 - 30	1.18 (1.14, 1.21)	
≥30	1.10 (1.06, 1.14)	
**Type 2 diabetes mellitus**		
No	1.19 (1.15, 1.23)	0.005
Yes	1.32 (1.22, 1.44)	
**Hypertension**		
No	1.19 (1.15, 1.23)	0.151
Yes	1.23 (1.16, 1.30)	
**Smoking status**		
No	1.19 (1.15, 1.23)	<0.001
Yes	1.32 (1.26, 1.38)	
**eGFR (mL/min/1.73m²)**		
<60	1.21 (1.15, 1.28)	0.285
≥60	1.19 (1.15, 1.23)	
**LDL cholesterol (mg/dL)**		
<100	1.12 (1.07, 1.17)	0.002
≥100 - 116	1.15 (1.11, 1.19)	
>116 - 150	1.18 (1.14, 1.22)	
>150	1.21 (1.16, 1.25)	
**Dyslipidemia**		
No	1.19 (1.15, 1.23)	0.954
Yes	1.18 (1.09, 1.29)	

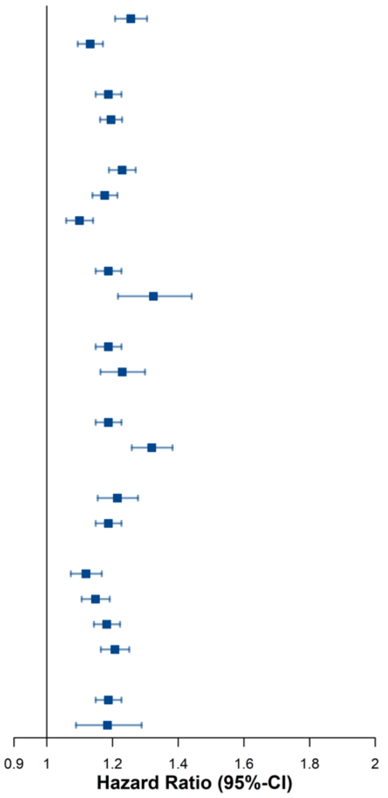

(D)

Subgroup	adj. HR (95%-CI)	p-value
**Age (years)**		
≤58 (median)	1.36 (1.29, 1.43)	<0.001
>58 (median)	1.18 (1.13, 1.24)	
**Sex**		
female	1.26 (1.20, 1.32)	0.670
male	1.27 (1.22, 1.32)	
**BMI (kg/m²)**		
<25	1.32 (1.26, 1.38)	<0.001
≥25 - 30	1.24 (1.19, 1.30)	
≥30	1.14 (1.08, 1.19)	
**Type 2 diabetes mellitus**		
No	1.26 (1.20, 1.32)	0.005
Yes	1.46 (1.30, 1.63)	
**Hypertension**		
No	1.26 (1.20, 1.32)	0.151
Yes	1.32 (1.23, 1.42)	
**Smoking status**		
No	1.26 (1.20, 1.32)	<0.001
Yes	1.45 (1.36, 1.54)	
**eGFR (mL/min/1.73m²)**		
<60	1.30 (1.21, 1.39)	0.285
≥60	1.26 (1.20, 1.32)	
**LDL cholesterol (mg/dL)**		
<100	1.16 (1.10, 1.23)	0.002
≥100 - 116	1.20 (1.15, 1.27)	
>116 - 150	1.25 (1.20, 1.31)	
>150	1.29 (1.23, 1.35)	
**Dyslipidemia**		
No	1.26 (1.20, 1.32)	0.954
Yes	1.26 (1.12, 1.41)	

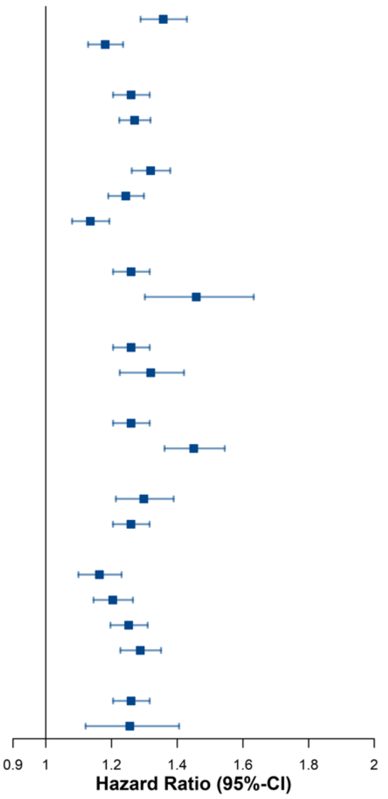

Figure S8. Association of hsCRP levels with MACE across clinical subgroups

Forest plot displaying the association of hsCRP with MACE in predefined clinical subgroups stratified by age (median), sex, BMI (<25 kg/m^2, ≥25-<30 kg/m^2, ≥30 kg/m^2), type 2 diabetes mellitus, hypertension, smoking status, eGFR (≥60 mL/min/1.73 m^2, <60 mL/min/1.73 m^2), LDL-C (<100 mg/dL, ≥100-116 mg/dL, >116-<150 mg/dL, ≥150 mg/dL) and dyslipidemia. Group-specific hazard ratios (HR) for hsCRP category change with 95% confidence intervals (CI) and p-values of interaction were derived from the multivariable Cox regression model (Model 1) that included all main effects and all hsCRP-by-subgroup interaction terms.

(A) HRs comparing individuals with hsCRP levels <1 mg/L to those with >3 mg/L.

(B) HRs comparing individuals with hsCRP levels <1 mg/L to those with 1-3 mg/L.

(C) HRs comparing individuals with hsCRP levels 1-3 mg/L to those with >3 mg/L.

(D) HRs comparing individuals with hsCRP levels ≥2 mg/L to those with <2 mg/L.

Abbreviations: hsCRP: high-sensitivity C-reactive protein; MACE: major adverse cardiovascular events; BMI: body mass index; eGFR: estimated glomerular filtration rate; LDL-C: low-density lipoprotein cholesterol

(A)

Subgroup	adj. HR (95%-CI)	p-value
**Age (years)**		
≤58 (median)	1.67 (1.42, 1.96)	0.025
>58 (median)	1.46 (1.28, 1.66)	
**Sex**		
female	1.55 (1.35, 1.77)	0.001
male	1.88 (1.68, 2.11)	
**BMI (kg/m²)**		
<25	1.66 (1.45, 1.89)	0.012
≥25 - 30	1.52 (1.33, 1.73)	
≥30	1.31 (1.13, 1.52)	
**Type 2 diabetes mellitus**		
No	1.55 (1.35, 1.77)	0.656
Yes	1.64 (1.22, 2.21)	
**Hypertension**		
No	1.55 (1.35, 1.77)	0.996
Yes	1.55 (1.26, 1.91)	
**Smoking status**		
No	1.55 (1.35, 1.77)	0.289
Yes	1.44 (1.21, 1.71)	
**eGFR (mL/min/1.73m²)**		
<60	1.63 (1.36, 1.96)	0.470
≥60	1.55 (1.35, 1.77)	
**LDL cholesterol (mg/dL)**		
<100	1.39 (1.18, 1.64)	0.056
≥100 - 116	1.46 (1.26, 1.69)	
>116 - 150	1.54 (1.34, 1.76)	
>150	1.66 (1.43, 1.92)	
**Dyslipidemia**		
No	1.55 (1.35, 1.77)	0.479
Yes	1.72 (1.25, 2.35)	

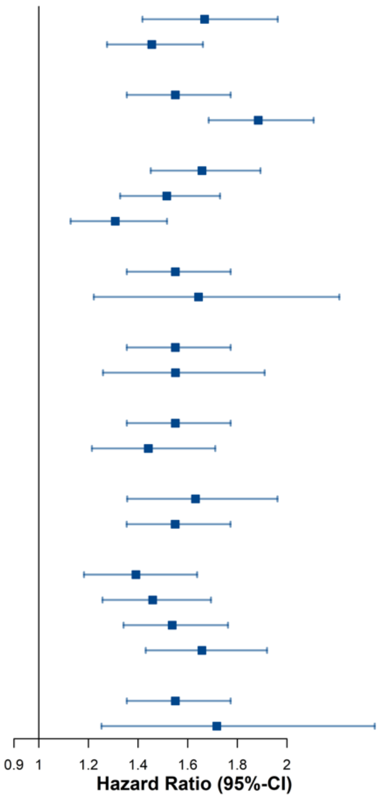

(B)

Subgroup	adj. HR (95%-CI)	p-value
**Age (years)**		
≤58 (median)	1.29 (1.19, 1.40)	0.025
>58 (median)	1.21 (1.13, 1.29)	
**Sex**		
female	1.24 (1.16, 1.33)	0.001
male	1.37 (1.30, 1.45)	
**BMI (kg/m²)**		
<25	1.29 (1.20, 1.37)	0.012
≥25 - 30	1.23 (1.15, 1.31)	
≥30	1.14 (1.06, 1.23)	
**Type 2 diabetes mellitus**		
No	1.24 (1.16, 1.33)	0.656
Yes	1.28 (1.10, 1.48)	
**Hypertension**		
No	1.24 (1.16, 1.33)	0.996
Yes	1.24 (1.12, 1.38)	
**Smoking status**		
No	1.24 (1.16, 1.33)	0.289
Yes	1.20 (1.10, 1.31)	
**eGFR (mL/min/1.73m²)**		
<60	1.28 (1.16, 1.40)	0.470
≥60	1.24 (1.16, 1.33)	
**LDL cholesterol (mg/dL)**		
<100	1.18 (1.09, 1.28)	0.056
≥100 - 116	1.21 (1.12, 1.30)	
>116 - 150	1.24 (1.16, 1.33)	
>150	1.29 (1.20, 1.38)	
**Dyslipidemia**		
No	1.24 (1.16, 1.33)	0.479
Yes	1.31 (1.12, 1.53)	

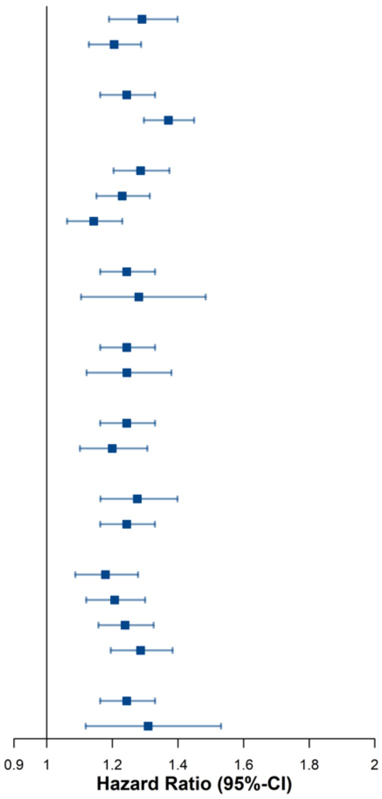

(C)

Subgroup	adj. HR (95%-CI)	p-value
**Age (years)**		
≤58 (median)	1.29 (1.19, 1.40)	0.025
>58 (median)	1.21 (1.13, 1.29)	
**Sex**		
female	1.25 (1.16, 1.33)	0.001
male	1.37 (1.30, 1.45)	
**BMI (kg/m²)**		
<25	1.29 (1.21, 1.38)	0.012
≥25 - 30	1.23 (1.15, 1.32)	
≥30	1.14 (1.06, 1.23)	
**Type 2 diabetes mellitus**		
No	1.25 (1.16, 1.33)	0.656
Yes	1.28 (1.11, 1.49)	
**Hypertension**		
No	1.25 (1.16, 1.33)	0.996
Yes	1.25 (1.12, 1.38)	
**Smoking status**		
No	1.25 (1.16, 1.33)	0.289
Yes	1.20 (1.10, 1.31)	
**eGFR (mL/min/1.73m²)**		
<60	1.28 (1.17, 1.40)	0.470
≥60	1.25 (1.16, 1.33)	
**LDL cholesterol (mg/dL)**		
<100	1.18 (1.09, 1.28)	0.056
≥100 - 116	1.21 (1.12, 1.30)	
>116 - 150	1.24 (1.16, 1.33)	
>150	1.29 (1.20, 1.39)	
**Dyslipidemia**		
No	1.25 (1.16, 1.33)	0.479
Yes	1.31 (1.12, 1.54)	

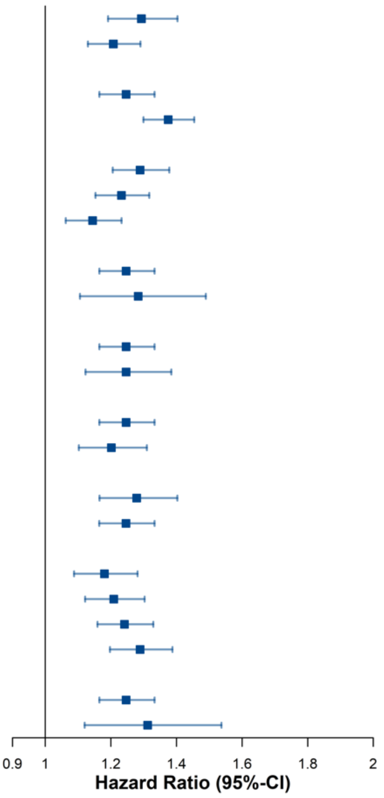

(D)

Subgroup	adj. HR (95%-CI)	p-value
**Age (years)**		
≤58 (median)	1.41 (1.27, 1.58)	0.025
>58 (median)	1.29 (1.18, 1.41)	
**Sex**		
female	1.34 (1.23, 1.47)	0.001
male	1.53 (1.42, 1.65)	
**BMI (kg/m²)**		
<25	1.41 (1.29, 1.54)	0.012
≥25 - 30	1.32 (1.21, 1.45)	
≥30	1.20 (1.08, 1.32)	
**Type 2 diabetes mellitus**		
No	1.34 (1.23, 1.47)	0.656
Yes	1.40 (1.14, 1.71)	
**Hypertension**		
No	1.34 (1.23, 1.47)	0.996
Yes	1.34 (1.17, 1.55)	
**Smoking status**		
No	1.34 (1.23, 1.47)	0.289
Yes	1.28 (1.14, 1.44)	
**eGFR (mL/min/1.73m²)**		
<60	1.39 (1.23, 1.57)	0.470
≥60	1.34 (1.23, 1.47)	
**LDL cholesterol (mg/dL)**		
<100	1.25 (1.12, 1.39)	0.056
≥100 - 116	1.29 (1.17, 1.43)	
>116 - 150	1.34 (1.22, 1.47)	
>150	1.41 (1.27, 1.55)	
**Dyslipidemia**		
No	1.34 (1.23, 1.47)	0.479
Yes	1.44 (1.16, 1.78)	

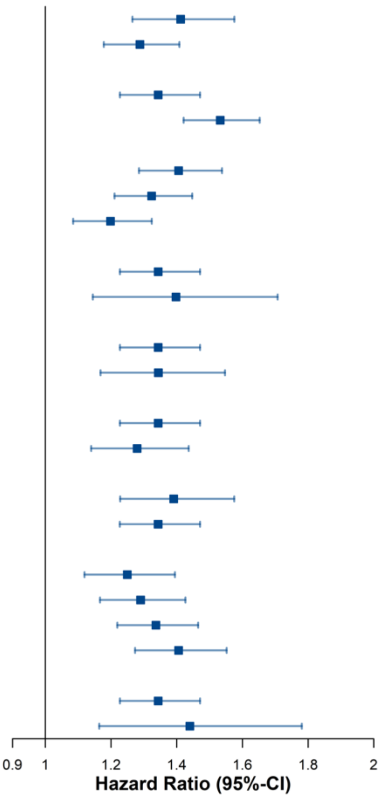

Figure S9. Association of hsCRP levels with CV death across clinical subgroups

Forest plot displaying the association of hsCRP with CV death in predefined clinical subgroups stratified by age (median), sex, BMI (<25 kg/m^2, ≥25-<30 kg/m^2, ≥30 kg/m^2), type 2 diabetes mellitus, hypertension, smoking status, eGFR (≥60 mL/min/1.73 m^2, <60 mL/min/1.73 m^2), LDL-C (<100 mg/dL, ≥100-116 mg/dL, >116-<150 mg/dL, ≥150 mg/dL) and dyslipidemia. Group-specific hazard ratios (HR) for hsCRP category change with 95% confidence intervals (CI) and p-values of interaction were derived from the multivariable Cox regression model (Model 1) that included all main effects and all hsCRP-by-subgroup interaction terms.

(A) HRs comparing individuals with hsCRP levels <1 mg/L to those with >3 mg/L.

(B) HRs comparing individuals with hsCRP levels <1 mg/L to those with 1-3 mg/L.

(C) HRs comparing individuals with hsCRP levels 1-3 mg/L to those with >3 mg/L.

(D) HRs comparing individuals with hsCRP levels ≥2 mg/L to those with <2 mg/L.

Abbreviations: hsCRP: high-sensitivity C-reactive protein; MACE: major adverse cardiovascular events; BMI: body mass index; eGFR: estimated glomerular filtration rate; LDL-C: low-density lipoprotein cholesterol

(A)

Subgroup	adj. HR (95%-CI)	p-value
**Age (years)**		
≤58 (median)	1.74 (1.63, 1.85)	<0.001
>58 (median)	1.44 (1.37, 1.52)	
**Sex**		
female	1.57 (1.49, 1.65)	<0.001
male	1.73 (1.66, 1.82)	
**BMI (kg/m²)**		
<25	1.70 (1.62, 1.78)	<0.001
≥25 - 30	1.55 (1.47, 1.63)	
≥30	1.42 (1.34, 1.51)	
**Type 2 diabetes mellitus**		
No	1.57 (1.49, 1.65)	0.389
Yes	1.66 (1.45, 1.89)	
**Hypertension**		
No	1.57 (1.49, 1.65)	0.048
Yes	1.70 (1.55, 1.85)	
**Smoking status**		
No	1.57 (1.49, 1.65)	0.799
Yes	1.58 (1.48, 1.70)	
**eGFR (mL/min/1.73m²)**		
<60	1.83 (1.69, 1.97)	<0.001
≥60	1.57 (1.49, 1.65)	
**LDL cholesterol (mg/dL)**		
<100	1.47 (1.38, 1.57)	0.010
≥100 - 116	1.53 (1.45, 1.62)	
>116 - 150	1.57 (1.49, 1.65)	
>150	1.60 (1.51, 1.69)	
**Dyslipidemia**		
No	1.57 (1.49, 1.65)	0.793
Yes	1.60 (1.39, 1.84)	

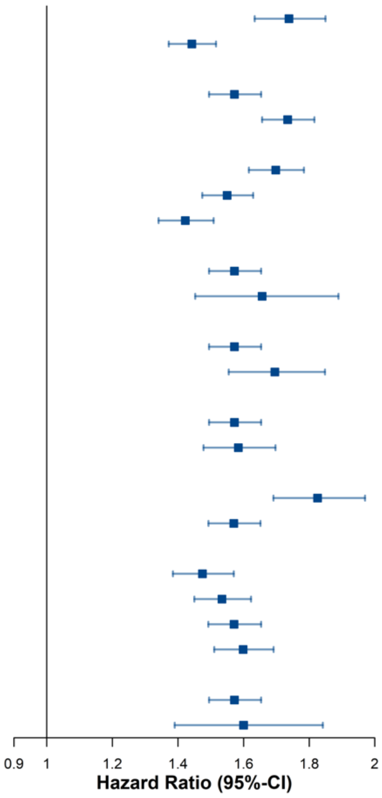

(B)

Subgroup	adj. HR (95%-CI)	p-value
**Age (years)**		
≤58 (median)	1.32 (1.28, 1.36)	<0.001
>58 (median)	1.20 (1.17, 1.23)	
**Sex**		
female	1.25 (1.22, 1.28)	<0.001
male	1.32 (1.29, 1.35)	
**BMI (kg/m²)**		
<25	1.30 (1.27, 1.33)	<0.001
≥25 - 30	1.24 (1.21, 1.28)	
≥30	1.19 (1.16, 1.23)	
**Type 2 diabetes mellitus**		
No	1.25 (1.22, 1.28)	0.389
Yes	1.29 (1.20, 1.37)	
**Hypertension**		
No	1.25 (1.22, 1.28)	0.048
Yes	1.30 (1.25, 1.36)	
**Smoking status**		
No	1.25 (1.22, 1.28)	0.799
Yes	1.26 (1.21, 1.30)	
**eGFR (mL/min/1.73m²)**		
<60	1.35 (1.30, 1.40)	<0.001
≥60	1.25 (1.22, 1.28)	
**LDL cholesterol (mg/dL)**		
<100	1.21 (1.18, 1.25)	0.010
≥100 - 116	1.24 (1.20, 1.27)	
>116 - 150	1.25 (1.22, 1.28)	
>150	1.26 (1.23, 1.30)	
**Dyslipidemia**		
No	1.25 (1.22, 1.28)	0.793
Yes	1.26 (1.18, 1.36)	

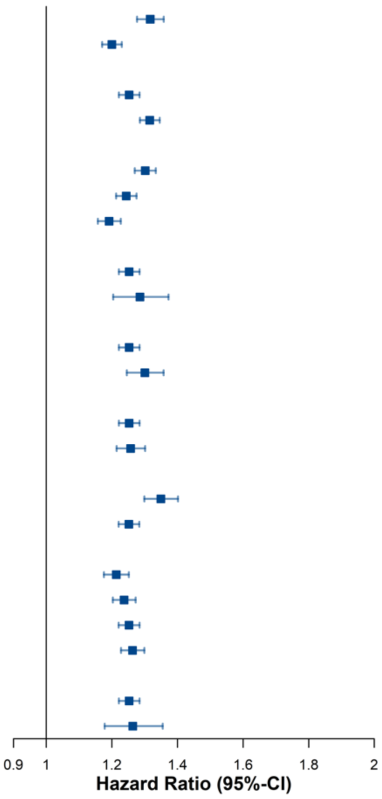

(C)

Subgroup	adj. HR (95%-CI)	p-value
**Age (years)**		
≤58 (median)	1.32 (1.28, 1.36)	<0.001
>58 (median)	1.20 (1.17, 1.23)	
**Sex**		
female	1.26 (1.22, 1.29)	<0.001
male	1.32 (1.29, 1.35)	
**BMI (kg/m²)**		
<25	1.30 (1.27, 1.34)	<0.001
≥25 - 30	1.25 (1.22, 1.28)	
≥30	1.19 (1.16, 1.23)	
**Type 2 diabetes mellitus**		
No	1.26 (1.22, 1.29)	0.389
Yes	1.29 (1.21, 1.38)	
**Hypertension**		
No	1.26 (1.22, 1.29)	0.048
Yes	1.30 (1.25, 1.36)	
**Smoking status**		
No	1.26 (1.22, 1.29)	0.799
Yes	1.26 (1.22, 1.30)	
**eGFR (mL/min/1.73m²)**		
<60	1.35 (1.30, 1.41)	<0.001
≥60	1.25 (1.22, 1.29)	
**LDL cholesterol (mg/dL)**		
<100	1.22 (1.18, 1.25)	0.010
≥100 - 116	1.24 (1.21, 1.28)	
>116 - 150	1.25 (1.22, 1.29)	
>150	1.27 (1.23, 1.30)	
**Dyslipidemia**		
No	1.26 (1.22, 1.29)	0.793
Yes	1.27 (1.18, 1.36)	

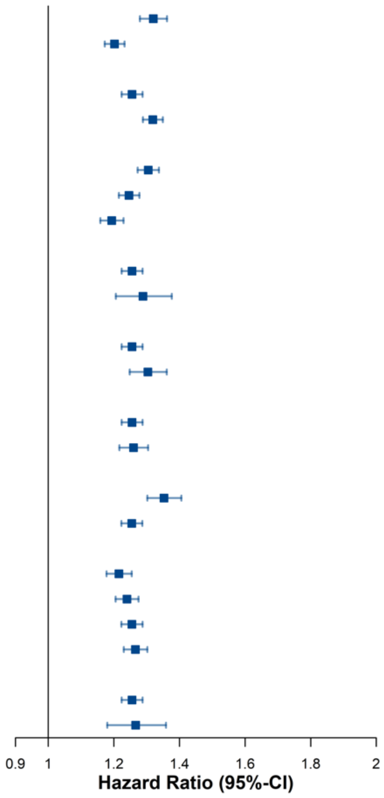

(D)

Subgroup	adj. HR (95%-CI)	p-value
**Age (years)**		
≤58 (median)	1.45 (1.39, 1.51)	<0.001
>58 (median)	1.28 (1.24, 1.32)	
**Sex**		
female	1.36 (1.31, 1.40)	<0.001
male	1.45 (1.41, 1.50)	
**BMI (kg/m²)**		
<25	1.43 (1.38, 1.48)	<0.001
≥25 - 30	1.34 (1.30, 1.39)	
≥30	1.27 (1.22, 1.32)	
**Type 2 diabetes mellitus**		
No	1.36 (1.31, 1.40)	0.389
Yes	1.41 (1.29, 1.54)	
**Hypertension**		
No	1.36 (1.31, 1.40)	0.048
Yes	1.43 (1.35, 1.51)	
**Smoking status**		
No	1.36 (1.31, 1.40)	0.799
Yes	1.36 (1.30, 1.43)	
**eGFR (mL/min/1.73m²)**		
<60	1.50 (1.43, 1.58)	<0.001
≥60	1.36 (1.31, 1.40)	
**LDL cholesterol (mg/dL)**		
<100	1.30 (1.25, 1.36)	0.010
≥100 - 116	1.33 (1.28, 1.39)	
>116 - 150	1.36 (1.31, 1.40)	
>150	1.37 (1.32, 1.43)	
**Dyslipidemia**		
No	1.36 (1.31, 1.40)	0.793
Yes	1.37 (1.25, 1.51)	

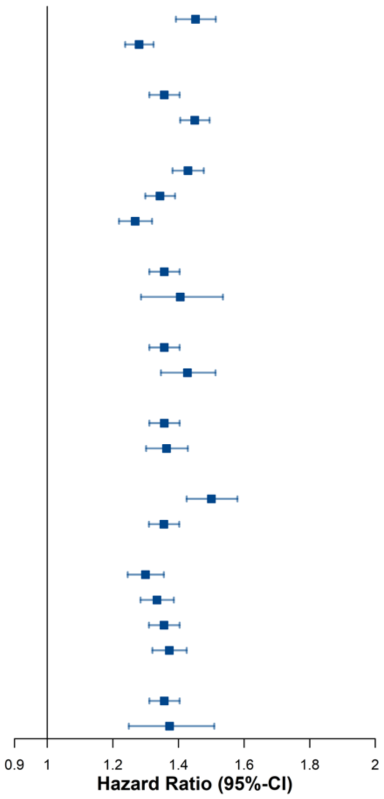

Figure S10. Association of hsCRP levels with all-cause death across clinical subgroups

Forest plot displaying the association of hsCRP with all-cause death in predefined clinical subgroups stratified by age (median), sex, BMI (<25 kg/m^2, ≥25-<30 kg/m^2, ≥30 kg/m^2), type 2 diabetes mellitus, hypertension, smoking status, eGFR (≥60 mL/min/1.73 m^2, <60 mL/min/1.73 m^2), LDL-C (<100 mg/dL, ≥100-116 mg/dL, >116-<150 mg/dL, ≥150 mg/dL) and dyslipidemia. Group-specific hazard ratios (HR) for hsCRP category change with 95% confidence intervals (CI) and p-values of interaction were derived from the multivariable Cox regression model (Model 1) that included all main effects and all hsCRP-by-subgroup interaction terms.

(A) HRs comparing individuals with hsCRP levels <1 mg/L to those with >3 mg/L.

(B) HRs comparing individuals with hsCRP levels <1 mg/L to those with 1-3 mg/L.

(C) HRs comparing individuals with hsCRP levels 1-3 mg/L to those with >3 mg/L.

(D) HRs comparing individuals with hsCRP levels ≥2 mg/L to those with <2 mg/L.

Abbreviations: hsCRP: high-sensitivity C-reactive protein; MACE: major adverse cardiovascular events; BMI: body mass index; eGFR: estimated glomerular filtration rate; LDL-C: low-density lipoprotein cholesterol

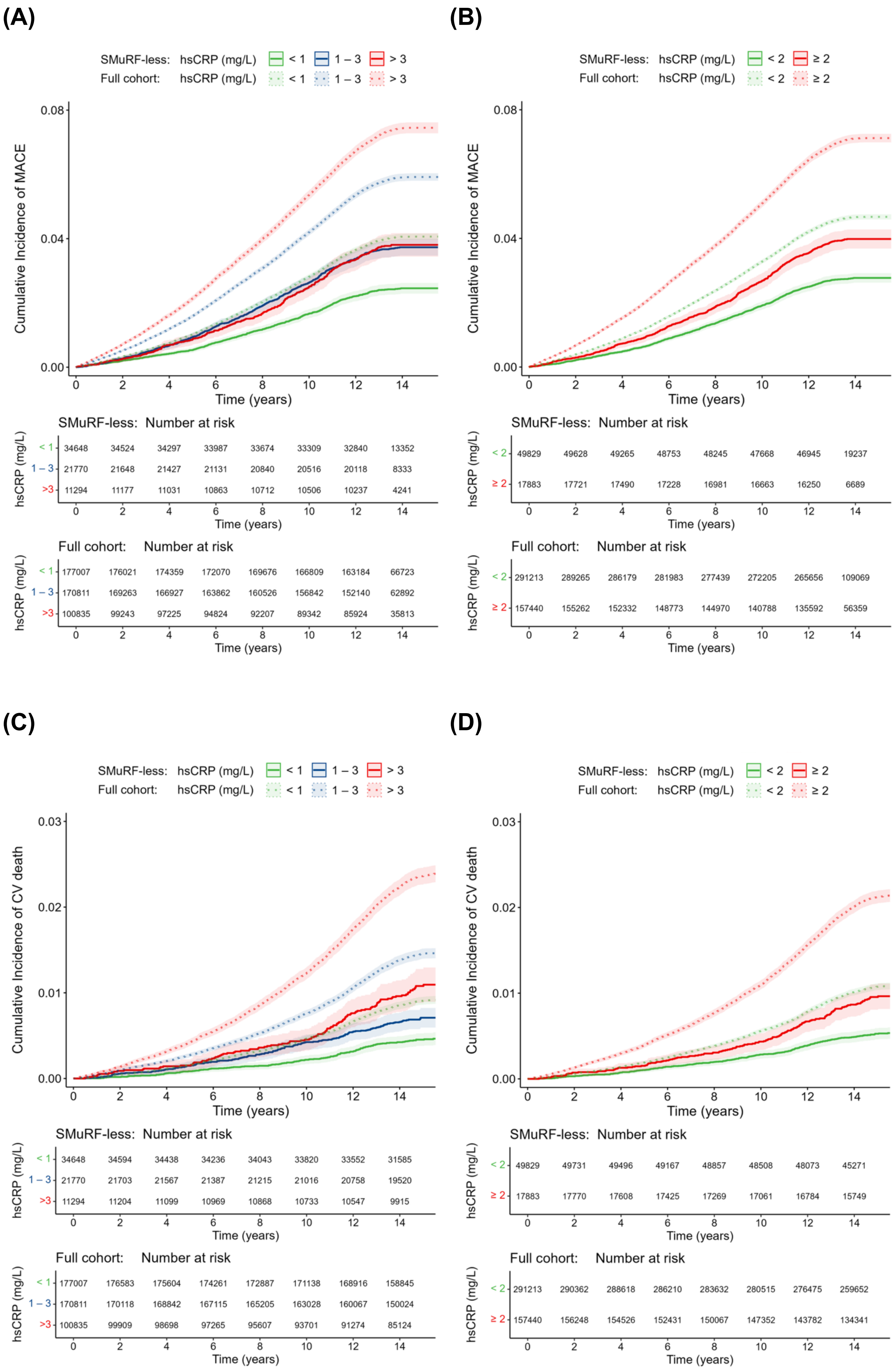
(A)
SMuRF-less: hsCRP (mg/L) < 1 1 – 3 > 3
Full cohort: hsCRP (mg/L) < 1 1 – 3 > 3
Cumulative Incidence of MACE
Time (years)
SMuRF-less: Number at risk
< 1 34648 34524 34297 33987 33674 33309 32840 13352
1 – 3 21770 21648 21427 21131 20840 20516 20118 8333
>3 11294 11177 11031 10863 10712 10506 10237 4241
Full cohort: Number at risk
< 1 177007 176021 174359 172070 169676 166809 163184 66723
1 – 3 170811 169263 166927 163862 160526 156842 152140 62892
>3 100835 99243 97225 94824 92207 89342 85924 35813
hsCRP (mg/L)
(B)
SMuRF-less: hsCRP (mg/L) < 2 ≥ 2
Full cohort: hsCRP (mg/L) < 2 ≥ 2
Cumulative Incidence of MACE
Time (years)
SMuRF-less: Number at risk
< 2 49829 49628 49265 48753 48245 47668 46945 19237
≥ 2 17883 17721 17490 17228 16981 16663 16250 6689
Full cohort: Number at risk
< 2 291213 289265 286179 281983 277439 272205 265656 109069
≥ 2 157440 155262 152332 148773 144970 140788 135592 56359
hsCRP (mg/L)
(C)
SMuRF-less: hsCRP (mg/L) < 1 1 – 3 > 3
Full cohort: hsCRP (mg/L) < 1 1 – 3 > 3
Cumulative Incidence of CV death
Time (years)
SMuRF-less: Number at risk
< 1 34648 34594 34438 34236 34043 33820 33552 31585
1 – 3 21770 21703 21567 21387 21215 21016 20758 19520
>3 11294 11204 11099 10969 10868 10733 10547 9915
Full cohort: Number at risk
< 1 177007 176583 175604 174261 172887 171138 168916 158845
1 – 3 170811 170118 168842 167115 165205 163028 160067 150024
>3 100835 99909 98698 97265 95607 93701 91274 85124
hsCRP (mg/L)
(D)
SMuRF-less: hsCRP (mg/L) < 2 ≥ 2
Full cohort: hsCRP (mg/L) < 2 ≥ 2
Cumulative Incidence of CV death
Time (years)
SMuRF-less: Number at risk
< 2 49829 49731 49496 49167 48857 48508 48073 45271
≥ 2 17883 17770 17608 17425 17269 17061 16784 15749
Full cohort: Number at risk
< 2 291213 290362 288618 286210 283632 280515 276475 259652
≥ 2 157440 156248 154526 152431 150067 147352 143782 134341
hsCRP (mg/L)

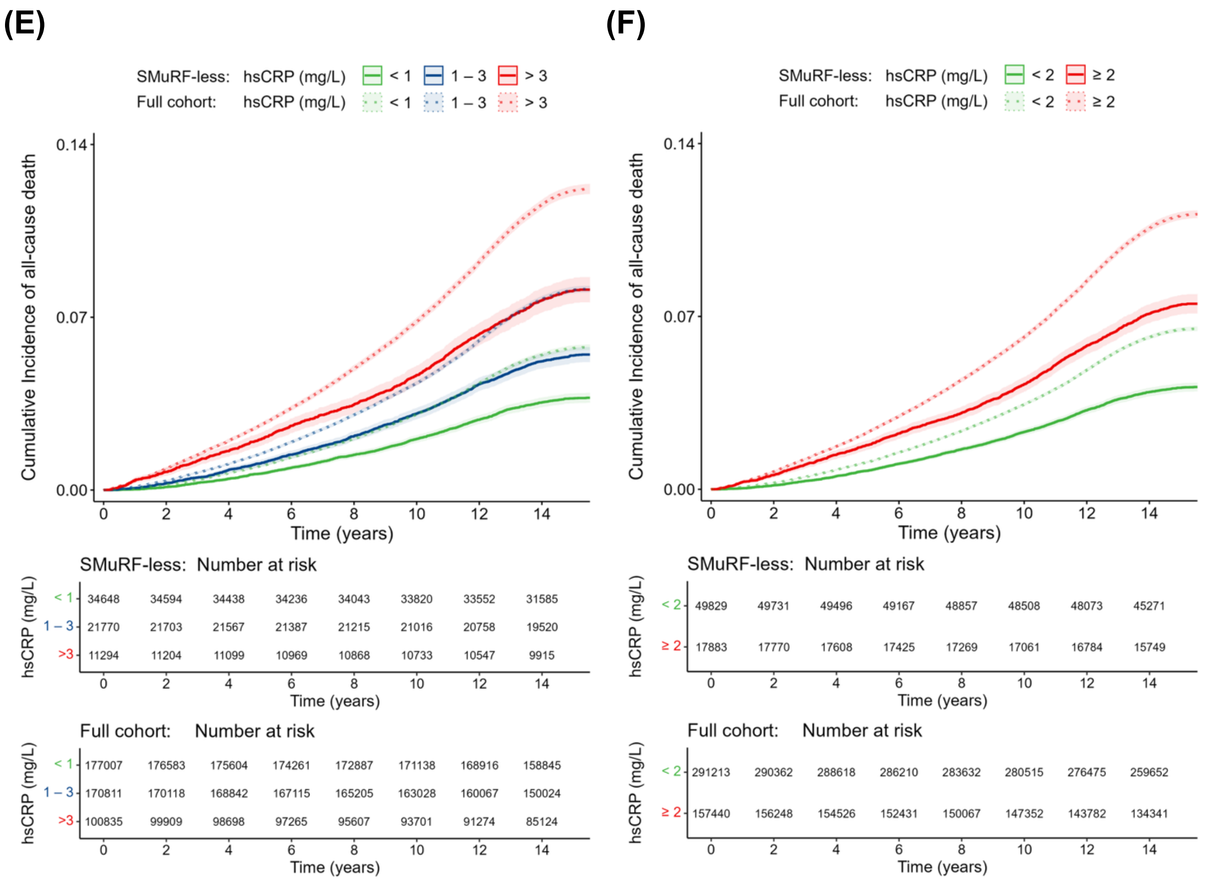

Figure S11. Association of hsCRP with CV outcomes in the full cohort and in individuals without standard modifiable CV risk factors (SMuRF)

Kaplan-Meier curves illustrating the cumulative incidence of MACE, CV death and all-cause death stratified by hsCRP levels in the full cohort and in individuals without SMuRF (“SMuRF-less”). The number at risk is displayed below the x-axis and shaded areas represent 95% confidence intervals.

(A) Incidence of MACE in the full cohort and in SMuRF-less individuals comparing levels of hsCRP <1 mg/L vs. 1-3 mg/L vs. >3 mg/L.

(B) Incidence of MACE in the full cohort and in SMuRF-less individuals comparing levels of hsCRP <2 mg/L vs. ≥2 mg/L.

(C) Incidence of CV death in the full cohort and in SMuRF-less individuals comparing levels of hsCRP <1 mg/L vs. 1-3 mg/L vs. >3 mg/L.

(D) Incidence of CV death in the full cohort and in SMuRF-less individuals comparing levels of hsCRP <2 mg/L vs. ≥2 mg/L.

(E) Incidence of all-cause death in the full cohort and in SMuRF-less individuals comparing levels of hsCRP <1 mg/L vs. 1-3 mg/L vs. >3 mg/L.

(F) Incidence of all-cause death in the full cohort and in SMuRF-less individuals comparing levels of hsCRP <2 mg/L vs. ≥2 mg/L.

All log-rank P-values <0.001.

Abbreviation: CV: cardiovascular; hsCRP: high-sensitivity C-reactive protein; MACE: major adverse cardiovascular events; SMuRF: standard modifiable cardiovascular risk factors

Supplementary references

1. Mach F, Baigent C, Catapano AL, Koskinas KC, Casula M, Badimon L, et al. 2019 ESC/EAS Guidelines for the management of dyslipidaemias: lipid modification to reduce cardiovascular risk. European Heart Journal. 2020;41(1):111-88.
2. Visseren FLJ, Mach F, Smulders YM, Carballo D, Koskinas KC, Back M, et al. 2021 ESC Guidelines on cardiovascular disease prevention in clinical practice. Eur Heart J. 2021;42(34):3227-337.
3. Marx N, Federici M, Schutt K, Muller-Wieland D, Ajjan RA, Antunes MJ, et al. 2023 ESC Guidelines for the management of cardiovascular disease in patients with diabetes. Eur Heart J. 2023;44(39):4043-140.
4. Group F-NBW. BEST (Biomarkers, EndpointS, and other Tools) Resource. BEST (Biomarkers, EndpointS, and other Tools) Resource. Silver Spring (MD) Bethesda (MD): Food and Drug Administration (US) National Institutes of Health (US); 2016.
5. Arnett DK, Blumenthal RS, Albert MA, Buroker AB, Goldberger ZD, Hahn EJ, et al. 2019 ACC/AHA Guideline on the Primary Prevention of Cardiovascular Disease: A Report of the American College of Cardiology/American Heart Association Task Force on Clinical Practice Guidelines. Circulation. 2019;140(11):e596-e646.
6. Pearson GJ, Thanassoulis G, Anderson TJ, Barry AR, Couture P, Dayan N, et al. 2021 Canadian Cardiovascular Society Guidelines for the Management of Dyslipidemia for the Prevention of Cardiovascular Disease in Adults. Canadian Journal of Cardiology. 2021;37(8):1129-50.
7. Ridker PM. A Test in Context: High-Sensitivity C-Reactive Protein. J Am Coll Cardiol. 2016;67(6):712-23.
8. Bellavia A, Murphy SA. Cox Regression Model in Clinical Research: Overview of Key Properties and Interpretation. Circulation. 2025;151(6):337-9.
9. Mazhar F, Faucon A-L, Fu EL, Szummer KE, Mathisen J, Gerward S, et al. Systemic inflammation and health outcomes in patients receiving treatment for atherosclerotic cardiovascular disease. European Heart Journal. 2024;45(44):4719-30.
10. Cook NR. Quantifying the added value of new biomarkers: how and how not. Diagnostic and Prognostic Research. 2018;2(1):14.
11. Vickers AJ, Cronin AM, Begg CB. One statistical test is sufficient for assessing new predictive markers. BMC Medical Research Methodology. 2011;11(1):13.
12. Pepe MS, Kerr KF, Longton G, Wang Z. Testing for improvement in prediction model performance. Statistics in Medicine. 2013;32(9):1467-82.
13. Koenig W, LöWel H, Baumert J, Meisinger C. C-Reactive Protein Modulates Risk Prediction Based on the Framingham Score. Circulation. 2004;109(11):1349-53.
14. Akaike H. Information Theory and an Extension of the Maximum Likelihood Principle. In: Parzen E, Tanabe K, Kitagawa G, editors. Selected Papers of Hirotugu Akaike. New York, NY: Springer New York; 1998. p. 199-213.
